# Identification and experimental validation of common genes associated with both pulmonary arterial hypertension and major depressive disorder

**DOI:** 10.3389/fpsyt.2025.1670519

**Published:** 2025-11-07

**Authors:** Yan Yan, Mohammad Ismail Hajary Sagor, Liang Chen, Huakan Lin, Gufeng Gao, Guili Lian, Liangdi Xie

**Affiliations:** 1 Department of Geriatrics, The First Affiliated Hospital of Fujian Medical University, Fujian, Fuzhou, China; 2 Fujian Hypertension Research Institute, The First Affiliated Hospital of Fujian Medical University, Fujian, Fuzhou, China; 3 Clinical Research Center for Geriatric Hypertension Disease of Fujian province, The First Affiliated Hospital of Fujian Medical University, Fujian, Fuzhou, China; 4 Branch of National Clinical Research Center for Aging and Medicine, The First Affiliated Hospital of Fujian Medical University, Fuzhou, Fujian, China; 5 Department of Geriatrics, National Regional Medical Center, Binhai Campus of the First Affiliated Hospital, Fujian Medical University, Fujian, Fuzhou, China

**Keywords:** pulmonary arterial hypertension, major depressive disorder, immune process, bioinformatics, validation

## Abstract

**Background:**

Pulmonary arterial hypertension (PAH) and major depressive disorder (MDD) frequently co-occur, worsening morbidity and mortality. The shared genetic and molecular substrates of this comorbidity remain unclear. This study investigated common differentially expressed genes (DEGs), convergent pathways, and candidate hub genes linking PAH and MDD.

**Methods:**

Gene-expression datasets for PAH (GSE113439, GSE53408) and MDD (GSE44593, GSE54564) were obtained from GEO. After standardization, DEGs were identified with Limma, and intersected across diseases while retaining concordant expression trends. Functional enrichment was performed using Gene Ontology (GO). A protein–protein interaction (PPI) network was built to prioritize hub genes (CytoHubba), followed by feature selection with LASSO regression and additional machine-learning validation. Immune-cell infiltration was profiled to assess shared immunological alterations. An experimental rat model of PAH exhibiting anxiety- and depression-like behaviors was established, and hub-gene expression was validated by qPCR.

**Results:**

Forty-two common DEGs with consistent directions were identified. Network analysis and LASSO converged on six candidate hub genes; among these, CHD8, DDX42, and EIF3D were further supported by machine-learning validation. Immune-infiltration analysis indicated dysregulated immune landscapes in both PAH and MDD. In PAH rats, anxiety- and depression-like behaviors were observed, and qPCR confirmed altered expression of CHD8, DDX42, and EIF3D consistent with in-silico findings.

**Conclusions:**

This integrative analysis highlights genetic and molecular links between PAH and MDD. CHD8, DDX42, and EIF3D emerge as candidate hub genes associated with the coexistence of these conditions, suggesting hypotheses for mechanistic follow-up and potential therapeutic targeting.

## Introduction

Pulmonary arterial hypertension (PAH) is a rare yet severe cardiovascular condition affecting about 1% of the global population (1). Characterized by a progressive increase in pulmonary vascular resistance and pulmonary artery pressure, PAH leads to debilitating symptoms like dyspnea, fatigue, syncope, and ultimately, right-sided heart failure (2). Despite advancements in targeted therapies, the survival rates at one and five years post-diagnosis remain modest at 86% and 61%, respectively (3).

PAH frequently co-occurs with major depressive disorder (MDD), a condition marked by a depressed mood, a decreased interest in activities, cognitive impairments, and physical symptoms such as sleep disturbances and appetite changes (4). Previous studies have indicated that PAH patients are more prone to MDD than healthy individuals (5), with prevalence rates of MDD ranging from 20% to 53% among those with PAH (6). Additionally, MDD adversely affects the quality of life and can negatively influence the prognosis of PAH, further reducing survival rates (7).

Both PAH and MDD share similar pathophysiological mechanisms, including dysregulation of the hypothalamic–pituitary–adrenal (HPA) axis, which is crucial in stress response and mood regulation. Chronic stress and cortisol dysregulation have been implicated in both conditions (4, 8). The HPA axis is hyperactive in depression, with increased corticotropin-releasing hormone production contributing significantly to this activity (9). Experimental findings suggest that angiotensin-converting enzyme 2 overexpression in the hypothalamus may reduce corticotropin-releasing hormone synthesis, offering protective effects against chronic hypoxia-induced pulmonary hypertension in mice (8). Additionally, imaging studies have shown significant gray matter damage and alterations in brain regions such as the hippocampus, amygdala, and temporal lobe in PAH patients, providing a structural basis for the mood disorders observed in these individuals (10).

Moreover, PAH is associated with elevated peripheral serotonin levels, implicating serotonin’s role in the pathophysiology of both PAH and depression. Although selective serotonin reuptake inhibitors are commonly used to treat depression by enhancing serotonin receptor activation via blocking its reuptake (11), their use in PAH patients has been linked with increased mortality and clinical worsening (12, 13). Thus, understanding the shared mechanisms between PAH and MDD as well as identifying novel therapeutic targets are crucial.

This study aimed to explore the biological underpinnings of PAH and MDD coexistence by identifying a common genetic signature through various bioinformatics tools, including differential gene expression analysis, PPI networks, and interaction network analyses. We also developed a PAH model to validate the association between negative emotions and identified hub genes through behavioral testing and quantitative polymerase chain reaction (qPCR) assays, providing new insights into the pathogenesis of both PAH and MDD.

## Materials and methods

### Data collection and preprocessing

**Figure 1:**
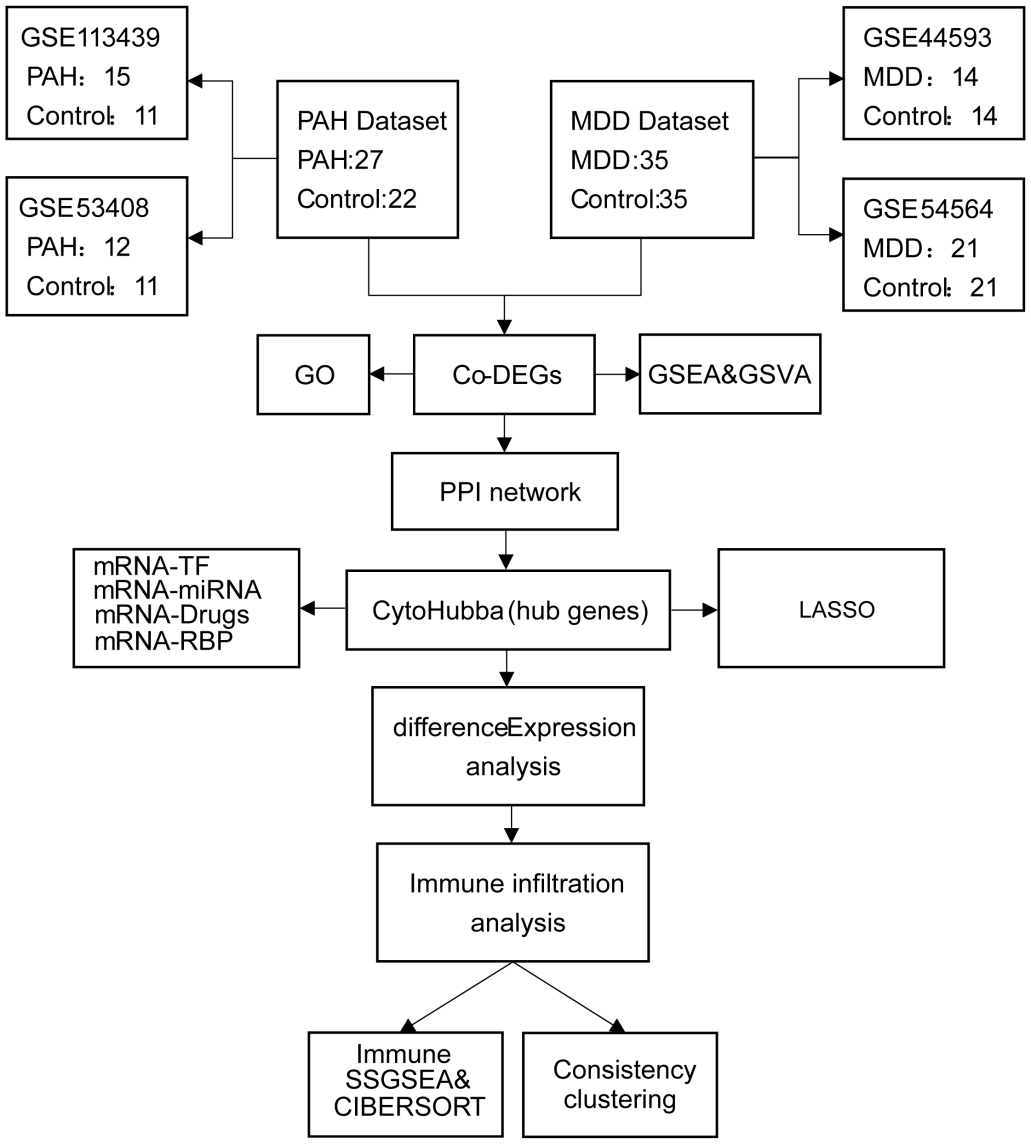
Flowchart of the study design. PAH, pulmonary arterial hypertension; MDD, major depressive disorder; Co-DEGs, common differentially expressed genes; PPI, protein–protein interaction; GO, gene ontology; GSEA, gene set enrichment analysis; TF, transcription factor.

The PAH datasets GSE113439 (14) and GSE53408 (15), together with the MDD datasets GSE44593 and GSE54564 (16), were obtained from the Gene Expression Omnibus (GEO) database (https://www.ncbi.nlm.nih.gov/geo/). Both PAH datasets consist of fresh-frozen human lung tissues from PAH patients and non-diseased controls: GSE113439 (GPL6244) includes 15 PAH and 11 control samples, and GSE53408 (GPL6244) contains 12 PAH and 11 control samples. Both MDD datasets comprise amygdala tissues from patients with MDD and matched controls: GSE44593 (GPL570) includes 14 MDD and 14 control samples, and GSE54564 (GPL6947) includes 21 MDD and 21 control samples. All cases and controls within each dataset were derived from the same tissue type, with no inclusion of samples from other organs or mixed tissues. Dataset integration and batch-effect correction were performed using the “sva” package (17), and cross-study normalization was carried out with the “Normalize Between Arrays” function in the “limma” package (18). Data reproducibility and reliability were assessed by principal component analysis (PCA). Detailed information on the datasets (platforms, tissue sources, sampling sites, and clinical characteristics) is provided in **Supplementary Table S1**, and a schematic overview of the study design is presented in **Figure 1**.

### Identification of differentially expressed genes

To identify the DEGs between cases and controls in the datasets, we used the “limma” package in R. The selection criteria for DEGs included an adjusted *p* value (from the Benjamini–Hochberg method) of less than 0.05 and a log2 absolute fold change of greater than 0.5 (19, 20). These thresholds ensured a robust identification of genes significantly altered in expression, minimizing false discoveries.

For visualization of the expression patterns and the significant changes, heatmaps were generated using the “heatmap” package, and volcano plots were constructed with the “ggplot2” package.

Additionally, a Venn diagram was employed to identify common differentially expressed genes (Co-DEGs) between the PAH and MDD datasets. This approach helps to pinpoint the genes that are consistently altered in both conditions, suggesting potential shared molecular pathways or mechanisms.

### Protein–protein interaction network construction

The protein–protein interaction (PPI) network was constructed using the STRING database (https://string-db.org/) based on the previously identified Co-DEGs. To maximize coverage of potential interactions and avoid an overly sparse network, we applied a combined score threshold of ≥ 0.150. This cutoff allowed inclusion of interactions with at least moderate confidence, ensuring sufficient connectivity while retaining biological relevance.

Hub gene analysis was then carried out in Cytoscape using the CytoHubba plugin, which identifies key nodes that may play critical roles in disease biology. The top 10 hub genes were ranked according to multiple topological algorithms provided by CytoHubba, including Maximum Neighborhood Component, Density of Maximum Neighborhood Component, and Maximal Clique Centrality.

### Gene regulatory network analysis

To elucidate the regulatory dynamics of hub genes, we employed the miRDB database (www.mirdb.org) to construct mRNA–miRNA regulatory networks. For mRNA–RNA-binding protein (RBP) interactions, we accessed the ENCORI database. Additionally, the CHIPBase database (https://rna.sysu.edu.cn/chipbase/) and the hTFtarget database (https://guolab.wchscu.cn/hTFtarget/#!/) provided insights into mRNA–transcription factor (TF) interaction networks. Pharmacological connections involving hub genes were explored using the Comparative Toxicogenomics Database (http://ctdbase.org/) to identify potential mRNA–drug interactions. These networks were visualized using Cytoscape software to map out the intricate relationships. Functional correlations among genes were analyzed using Friends analysis, which is a functional similarity analysis, employing the “GOSemSim” R package to calculate these relationships and identify key DEGs.

### Gene ontology analysis

To gain insights into the functional roles of the hub genes, we performed GO enrichment analyses using the “clusterProfiler” package (version 3.14.3). The analyses evaluated the genes across three main GO categories: molecular function (MF), cellular component (CC), and biological process (BP). To determine significance, we set thresholds for both the adjusted *p*-value and q-value at less than 0.05, ensuring the identification of highly relevant biological attributes associated with the hub genes.

### Hub−gene evaluation and ROC analysis

Hub-gene expression was assessed across four GEO datasets (PAH: GSE113439, GSE53408; MDD: GSE44593, GSE54564). No additional independent external validation cohort was available. Case–control differences were visualized with boxplots, and discriminative performance was evaluated using ROC analysis, with AUC values and 95% confidence intervals reported. Sample sizes, array platforms, and tissue sources for each dataset are provided in **Supplementary Table S1**.

### Gene set enrichment analysis

GSEA is a method used to determine if a predefined set of genes shows statistically significant, concordant differences between two biological states. For our analysis, we utilized the “c2.cp.all.v2022.1.Hs.symbols.gmt” gene set from the Molecular Signatures Database, which is accessible at https://www.gsea-msigdb.org/gsea/msigdb. We conducted GSEA using the “clusterprofiler” software package. The analysis was configured to identify gene sets enriched at a significance level of *p*<0.05 (21, 22).

### Gene set variation analysis

GSVA is an unsupervised method used to convert gene expression data from multiple samples into matrices of pathway activation scores. This approach helps to assess the enrichment of specific biological pathways. For our analysis, we used the “c2.cp.v2022.1.Hs.symbols” gene set from the Molecular Signatures Database, available at https://www.gsea-msigdb.org/gsea/msigdb. The differential pathway activity between disease and control groups was analyzed using the “limma” package, with significance determined by an adjusted *p*-value threshold of <0.01.

### Consensus clustering

Consensus clustering, a resampling-based approach, was utilized to explore cluster formation via the k-means algorithm. We employed the “Consensus Cluster Plus” package to categorize disease subtypes. Our evaluation included up to 10 potential categories, performing 100 iterations for each cluster number (k). For the clustering process, we selected the Partitioning Around Medoids algorithm paired with the Euclidean distance metric. To validate the differential expression of pivotal genes among the identified clusters, box plots were generated and analyzed.

### Immune infiltration and correlation analysis

To estimate immune cell proportions in the PAH and MDD samples, we applied the single-sample gene set enrichment analysis (ssGSEA) algorithm, using 28 predefined gene sets (23) that represent diverse immune cell types, including CD8+ T cells, dendritic cells, macrophages, and regulatory T cells. The “GSVA” R package was employed to compute the infiltration levels of these cells in each sample. Box plots were then used to compare the proportions of immune cells between the two datasets. Additionally, Spearman’s rank correlation analysis, facilitated by the “ggplot2” package, was conducted to assess the correlation between the infiltrating immune cells. This analysis also included the creation of scatter plots to visually explore the relationships between immunocytes and hub genes. As a complementary deconvolution approach, we used CIBERSORT (http://cibersort.stanford.edu/) to estimate relative immune cell proportions based on the LM22 signature, which consists of 547 genes distinguishing 22 human hematopoietic phenotypes, including seven T-cell subsets, naïve and memory B cells, plasma cells, NK cells, and various myeloid populations (24).

### Diagnostic model construction

We performed differential expression analysis and GSEA on samples from a combined dataset to identify genes commonly associated with PAH and MDD. Following this, we developed diagnostic models using the Logistic-Least Absolute Shrinkage and Selection Operator (Logistic-LASSO) technique, utilizing the “glmnet” package with a seed set to 2020 and the family parameter set to “binomial.” The predictive accuracy of our model was assessed through receiver operating characteristic (ROC) curve analysis.

### Animals

This study was approved by the Animal Welfare and Ethics Committee of Fujian Medical University (Approval No.: FJMU IACUC 2021-0387) and conducted in accordance with the ARRIVE guidelines. Male Sprague–Dawley rats (8 weeks old) were obtained from Shanghai SLACCAS Laboratory Animal Co., Ltd. (Certificate No.: SCXK 2012-0002) and housed under standard laboratory conditions with free access to food and water. Animals were randomly assigned (computer-generated sequence) to either the PAH group or the control group (n = 6 per group). Investigators responsible for behavioral testing and data analysis were blinded to group allocation.

Pulmonary arterial hypertension (PAH) was induced by a single intraperitoneal injection of monocrotaline (MCT; Sigma Aldrich, 30 mg/kg), while control rats received an equal volume of saline. Although a 60 mg/kg MCT dose is widely used to generate robust PAH within 3–4 weeks, it is associated with systemic toxicity, early right heart failure, and high mortality, which complicate longitudinal and behavioral assessments. In contrast, previous studies have shown that a lower dose of 30 mg/kg elevates right ventricular systolic pressure (RVSP) and the right ventricular hypertrophy index (RVHI), while producing lower lethality and allowing stable subchronic observations over 4–8 weeks (25–27). Based on this evidence, we selected 30 mg/kg to establish a reproducible, moderately severe PAH phenotype suitable for subsequent behavioral and molecular analyses.

To avoid the influence of invasive procedures on behavior, all behavioral tests were conducted during week 4 after injection. Immediately thereafter, right-heart catheterization was performed to measure RVSP, and RVHI was calculated as RV/(LV + S) to confirm model induction (25, 28). Lung tissue (right lower lobe) was snap-frozen in liquid nitrogen for subsequent qPCR analysis (25).

### Anxiety-like and depression-like behaviors

Behavioral testing was performed during week 4 after monocrotaline or saline injection, following a fixed sequence designed to minimize cross-test carryover and anxiogenic effects: Sucrose Preference Test (SPT) on Days 26–28 (48h adaptation with bottle switching, followed by 24h food/water deprivation and a 1h test), Open Field Test (OFT) on Day 29 (5min session), and Elevated Plus Maze (EPM) on Day 30 (5min session). Tests were spaced by ≥24 h and conducted during the light phase (09:00–12:00). Animals were habituated to the testing room for 30min before each assay, and all apparatuses were sanitized with 70% ethanol between animals. The order was chosen because SPT is minimally stressful and non-invasive for assessing anhedonia, OFT induces moderate novelty- and light-related anxiety, and EPM is the most anxiogenic due to elevation and open arms; therefore, EPM was performed last (29–32).

Sucrose Preference Test (SPT) — Anhedonia was assessed as described (33). Rats were habituated to 1% sucrose in two 150-mL bottles for 24h, followed by 24h with one bottle of water and one of 1% sucrose. After an additional 24-h food and water deprivation, rats were given 1h access to both bottles, with bottle positions switched halfway to avoid side bias. Intake (by weight) was recorded, and sucrose preference was calculated as [sucrose intake/total intake] × 100%. After SPT, animals were returned to ad libitum food and water before proceeding to the next assays.

Open Field Test (OFT) — Locomotor activity and anxiety-like behavior were assessed in a 100cm × 100cm arena with 40-cm walls (5-min session), recorded using an infrared camera (Model: TA-758RP) (34). Primary outcomes included total distance traveled, time spent in the center, and distance traveled in the center. Reduced center exploration was taken as an indicator of elevated anxiety.

Elevated Plus Maze (EPM) — Anxiety-like behavior was further evaluated in a 5-min session recorded from above (35). After 30min of acclimation to the testing room, each rat was placed in the maze center facing an open arm. A blinded investigator recorded the time spent in open arms and the number of open-arm entries. Reduced exploration of open arms was considered a sign of increased anxiety.

Where applicable, recovery intervals were provided between tests (SPT → OFT → EPM) to reduce stress and fatigue. Behavioral scoring for all tasks was performed by an investigator blinded to treatment.

### RNA extraction and qPCR

Total RNA was extracted from the right lung using the FastPure Cell/Tissue Total RNA Isolation Kit V2 (Vazyme). cDNA was synthesized with HiScript II Q RT SuperMix for qPCR (+gDNA wiper) (Vazyme). qPCR was carried out with ChamQ SYBR qPCR Master Mix on a LightCycler^®^ 96 system (Roche) in 20 µL reactions. Negative controls (NTC and –RT) were included, and melt-curve analysis (65–95 °C) confirmed single, specific products. Primer efficiencies ranged from 90–110% (R² > 0.99).

Gene expression was normalized to β-actin and calculated by the 2^−ΔΔCt^ method, with saline controls as the calibrator. Each group included six biological replicates, with triplicate technical replicates per sample. Data are presented as mean ± SEM. Statistical tests (independent-samples t-test or Mann–Whitney U when assumptions were violated) are specified in the Results and figure legends.

The primer sequences used for the qPCR:

CHD8: forward primer 5′-AGTGACGAGAAGGAAGA-3′
reverse primer 5′-GGGAATCCATCTTGGGACATAG-3′
EIF3D: forward primer 5′-CAACAAGCAGGTCATCCGAGTCTAC-3
reverse primer 5′-CCTCCTCTTCCTCCTCATCCTCTTC-3′
DDX42: forward primer 5′-CCCAAGGAGTCAACAACAC-3′
reverse primer 5′-ATGACGGCTACTGCTTTCT-3′
β−actin: forward primer 5′−CGCGAGTACAACCTTCTTGC−3′,
reverse primer 5′−CCTTCTGACCCATACCCACC−3′

### Statistical analysis

Bioinformatics analyses were performed in R, while animal experiment data were analyzed using GraphPad Prism 9.5 (GraphPad Software). Data are expressed as mean ± SEM. Between-group comparisons of MCT-induced PAH indices, behavioral outcomes, and qPCR results were evaluated using two-tailed independent-samples t-tests. Statistical significance was defined as P < 0.05. Assumptions of normality and homogeneity of variance were tested; when violated, nonparametric alternatives were applied (Mann–Whitney U for between-group comparisons, Wilcoxon signed-rank for paired data).

## Results

### Identification of common DEGs between PAH and MDD

**Figure 2:**
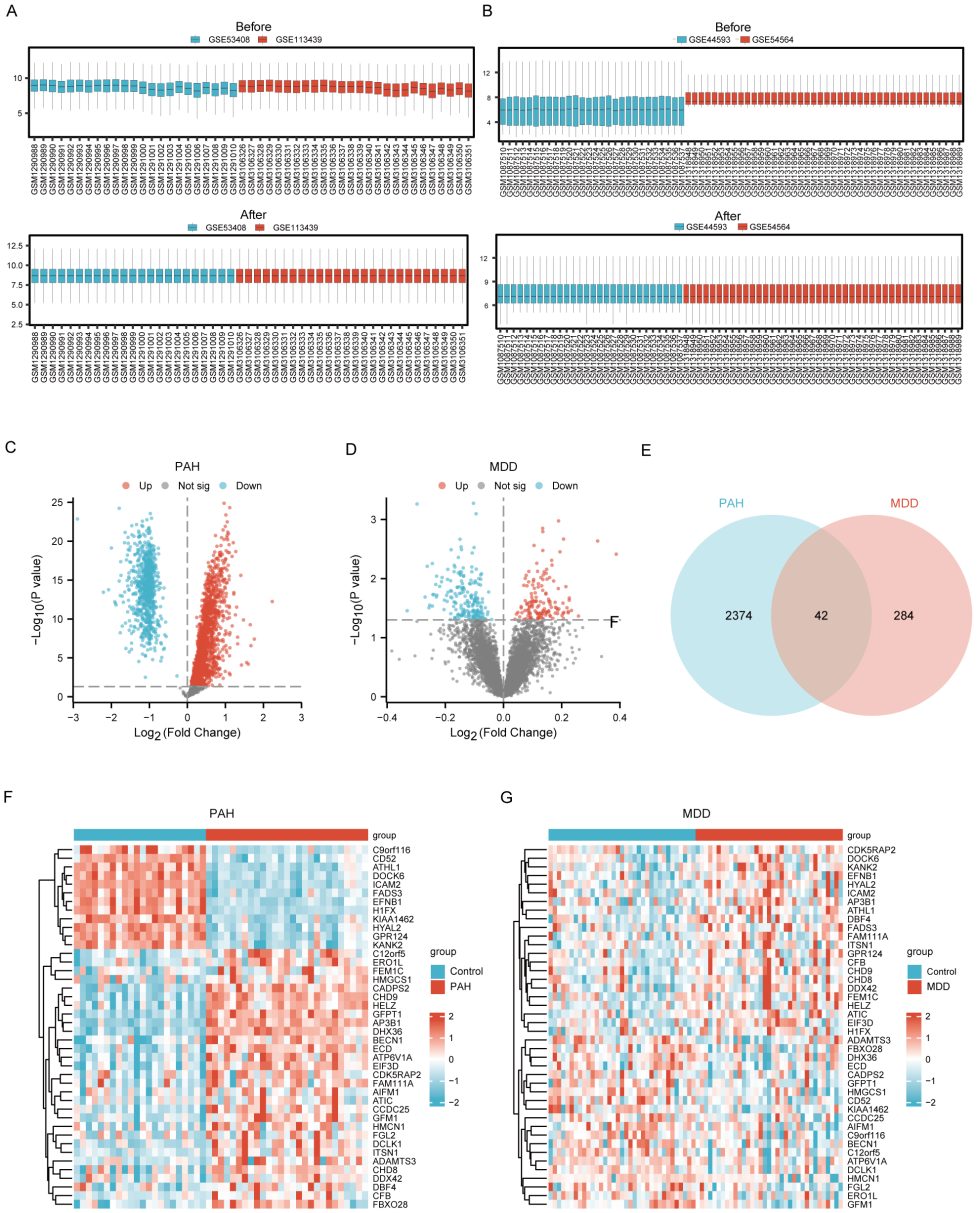
Identification of Common DEGs between PAH and MDD. **(A)** Raw expression matrix of the PAH dataset (Before) and Normalized expression matrix of the PAH dataset (After). **(B)** Raw expression matrix of the MDD dataset (Before) and Normalized expression matrix of the MDD dataset (After). **(C, D)**. Volcano plots of DEGs between the disease and control group in the PAH dataset **(C)** and MDD dataset **(D)**. These plots highlight significant DEGs with a fold change on the x-axis and the negative logarithm of the *p*-value on the y-axis, identifying genes significantly upregulated or downregulated. **(E)** A Venn diagram illustrating the overlap of DEGs between the PAH and MDD datasets, with DEGs that meet a significance threshold of a *p*<0.05. **(F, G)** Heatmaps of the 42 co-DEGs between the disease and control groups for the PAH dataset **(F)** and MDD dataset **(G)**.

The expression matrices from the PAH datasets (GSE53408, GSE113439) and the MDD datasets (GSE44593, GSE54564) were normalized. The resulting box plots displayed straight lines, indicating the distribution trends (**Figures 2A, B**).

Using the Limma R method, the analysis of the GSE53408 and GSE113439 datasets identified 2416 DEGs in the combined PAH dataset, with 1663 upregulated and 753 downregulated genes. Additionally, in the MDD dataset, a total of 326 DEGs were discovered, consisting of 136 upregulated and 190 downregulated genes. The volcano plots displaying the DEGs for both PAH and MDD are presented in **Figure 2C** and **Figure 2D**, respectively. The Venn diagram analysis revealed 42 DEGs common to both conditions (**Figure 2E**). Heatmaps showcasing these 42 common DEGs in both the PAH and MDD datasets are depicted in **Figures 2F, G** (**Supplementary Table S2**).

### GSEA and GSVA results of the PAH and MDD datasets

GSEA and GSVA were performed on both disease patients and healthy controls within the PAH and MDD datasets to uncover deeper biological insights into the behavior of DEGs.

**Figure 3:**
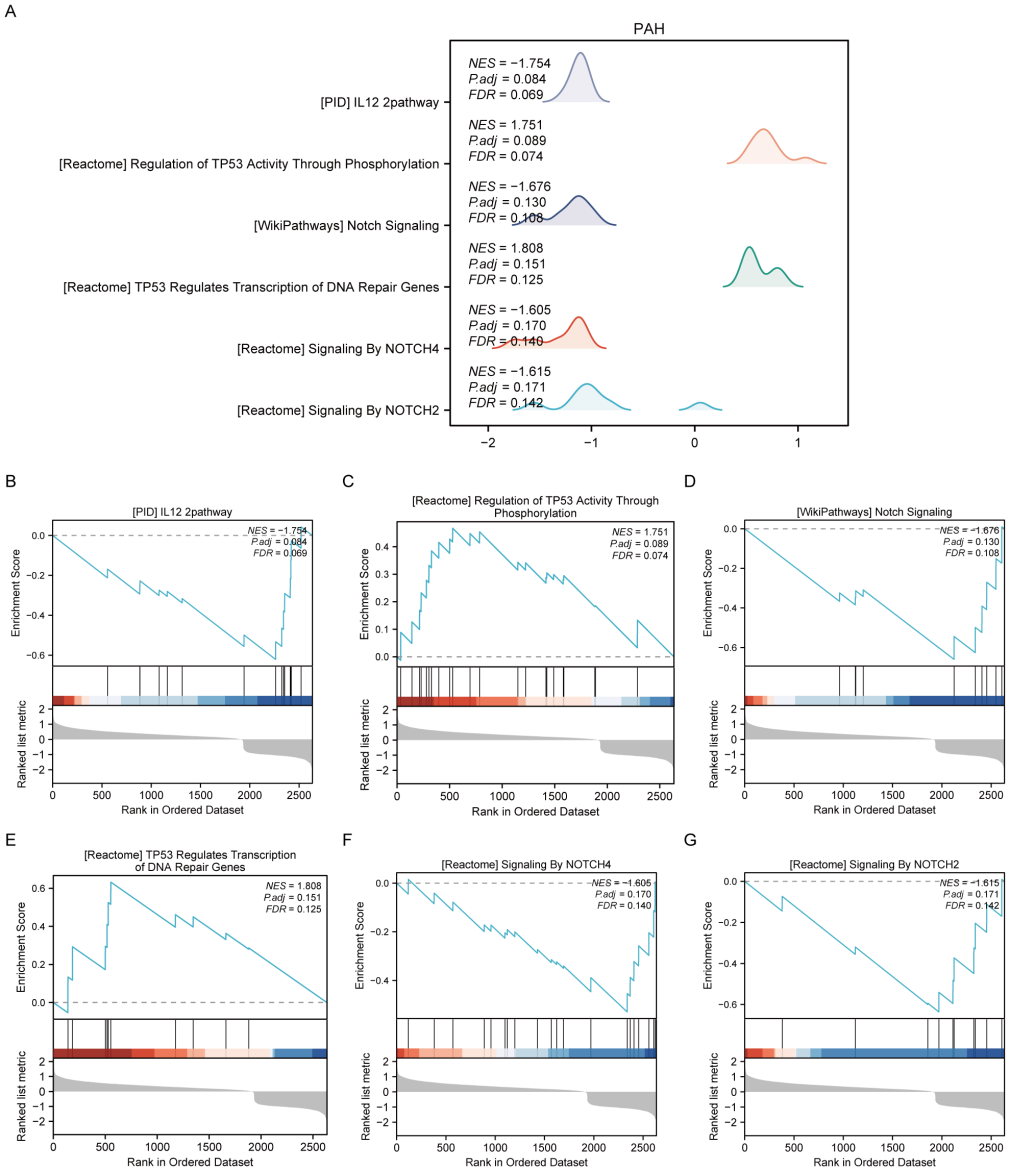
Results of gene set enrichment analysis (GSEA) of the PAH dataset. **(A)** Summary of the overall findings from GSEA, indicating which Reactome pathways were significantly enriched in the PAH dataset. **(B)** Genes significantly enriched in the “PID IL12 2PATHWAY,” revealing involvement in immune response modulation. **(C)** Enrichment in the “REGULATION OF TP53 ACTIVITY THROUGH PHOSPHORYLATION,” highlighting the pathway’s role in cell cycle control and apoptosis. **(D)** Enrichment in the “NOTCH SIGNALING” pathway, detailing gene enrichment that affects cell differentiation processes. **(E)** Enrichment in the “TP53 REGULATES TRANSCRIPTION OF DNA REPAIR GENES,” emphasizing the pathway’s importance in genomic stability. **(F, G)** Enrichment of genes in “SIGNALING BY NOTCH4” and “SIGNALING BY NOTCH2,” respectively, pointing out their specific roles in cellular development and fate determination.

For the PAH dataset, GSEA revealed that DEGs in the disease group (PAH/control) are significantly enriched in several key pathways. These include the interleukin-12 (IL-12) signaling pathway, the regulation of tumor protein TP53 activity through phosphorylation, Notch signaling, TP53-regulated transcription of DNA repair genes, signaling by NOTCH4, and signaling by NOTCH2 (**Figure 3**, **Supplementary Table S3**). Furthermore, GSVA of the PAH/control datasets identified that 10 gene sets, such as the PID p38 gamma delta pathway, showed statistical significance (*p*<0.05) among the PAH/control samples in the PAH dataset (**Supplementary Figure S1**, **Supplementary Table S5**).

**Figure 4:**
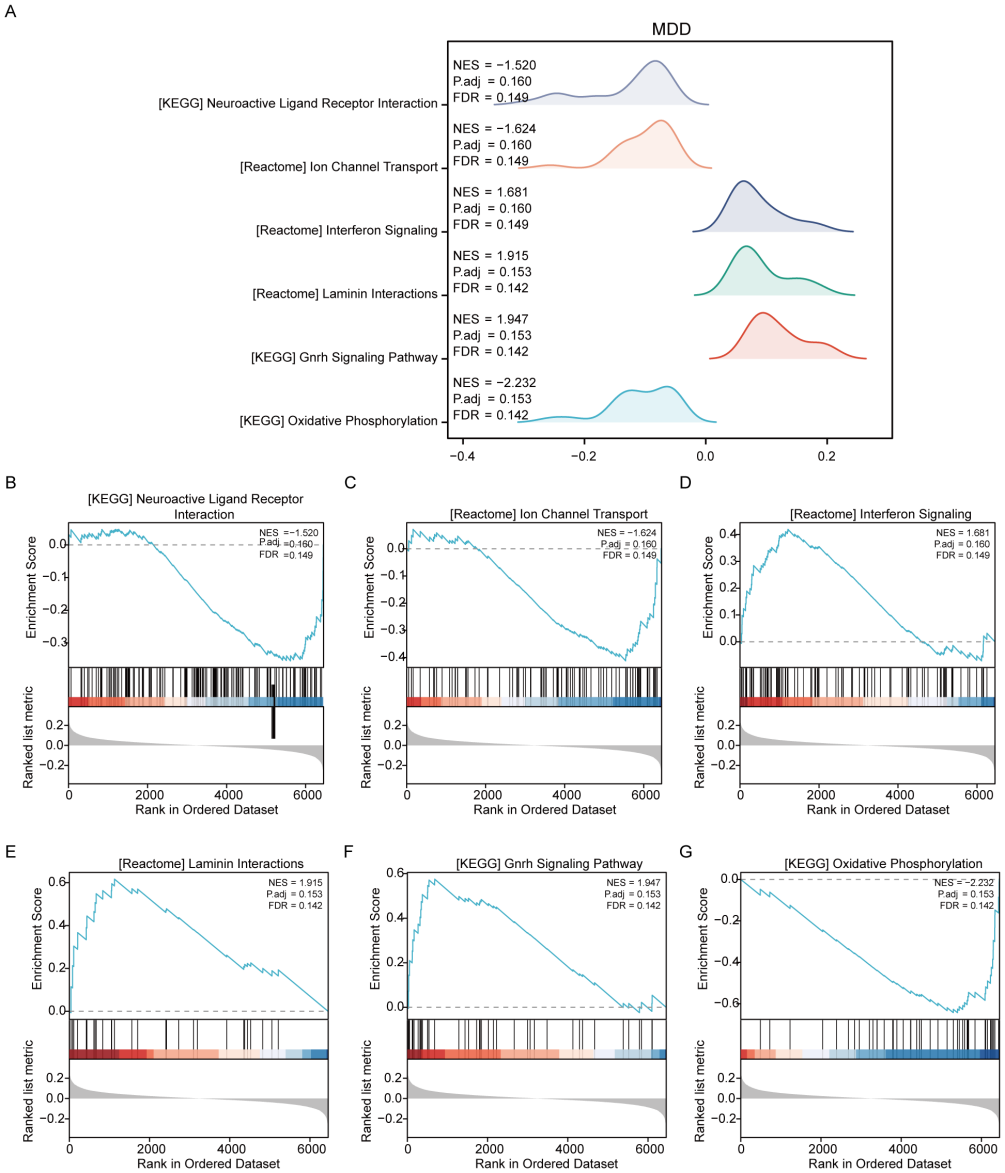
Results of gene set variation analysis of the MDD dataset. **(A)** Overview of the enriched Reactome pathways discovered in the MDD dataset through GSEA, setting the stage for detailed explorations. **(B)** Genes significantly enriched in the “NEUROACTIVE LIGAND-RECEPTOR INTERACTION” pathway, suggesting a major role in neurotransmitter dynamics, which are crucial for brain function and mood regulation. **(C)** Enrichment in “ION CHANNEL TRANSPORT,” highlighting its importance in neuronal excitability and signaling, factors that can influence depressive behaviors. **(D)** Significant gene involvement in “INTERFERON SIGNALING,” indicating potential links between immune response and psychiatric conditions like depression. **(E)** Genes enriched in “LAMININ INTERACTIONS,” which are essential for cell adhesion and integrity, impacting brain structural and synaptic functions. **(F)** Details of the “GNRH SIGNALING PATHWAY,” which is associated with the regulation of reproductive hormones that may also influence mood and emotional states. **(G)** Enrichment in “OXIDATIVE PHOSPHORYLATION,” a pathway crucial for energy metabolism, which has been implicated in the pathophysiology of depression due to energy dysregulation in brain cells.

For the MDD dataset, significant enrichment of genes was observed across various biological pathways when comparing disease groups to controls (MDD/control). Notable pathways that showed significant enrichment included neuroactive ligand–receptor interaction, ion channel transport, interferon signaling, laminin interactions, the gonadotropin-releasing hormone signaling pathway, and oxidative phosphorylation (**Figure 4**, **Supplementary Table S4**).

Additionally, GSVA was conducted on the MDD dataset to further compare the disease and control groups (MDD/control). The analysis identified that seven gene sets, including DNA mismatch repair, were statistically significant between the MDD and control groups within the MDD dataset (**Supplementary Figure S2**, **Supplementary Table S6**).

### Identification and external validation of candidate hub genes

**Figure 5:**
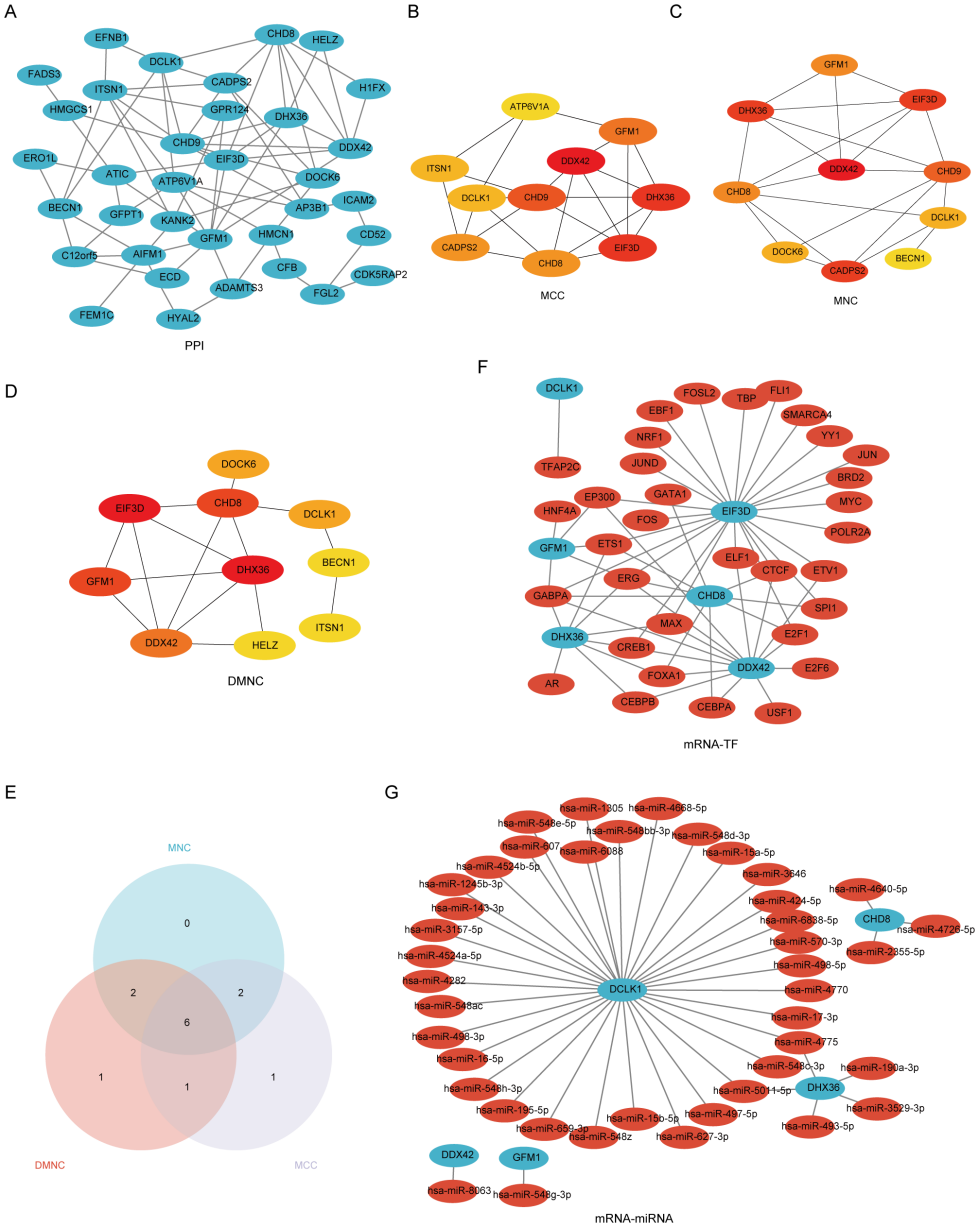
A comprehensive view of the interaction network of common differentially expressed genes (Co-DEGs) in the study of PAH and MDD. **(A)** The protein–protein interaction (PPI) network of Co-DEGs. This network illustrates how proteins encoded by these genes might interact with each other, suggesting potential functional collaborations or signaling cascades that are perturbed in both conditions. **(B–D)** The identification of hub genes within the PPI network using three different computational models. **(B)** Hub genes identified using the Matthews correlation coefficient (MCC) metric, which considers the correlation between gene pairs within the network. **(C)** Hub genes pinpointed by the maximal neighborhood component (MNC), focusing on genes with the largest and most significant local network connections. **(D)** Hub genes derived through differential metabolic network construction (DMNC), which identifies key genes based on their metabolic network roles. **(E)** The Venn diagram used to pinpoint the six hub genes common to both the PAH and MDD datasets, illustrating the overlap and suggesting genes of significant interest due to their potential shared roles in the pathophysiology of both diseases. **(F)** The mRNA–transcription factor (TF) regulatory network, maps out the interactions between target genes and their regulating TFs, providing insights into the gene regulation mechanisms altered in the diseases. **(G)** The mRNA-microRNA (miRNA) regulatory network. This network details the interactions between target genes and miRNAs, offering a look into how gene expression is post-transcriptionally regulated in the context of PAH and MDD.

The process of identifying and externally validating key genes that play a significant role in both PAH and MDD involved several sophisticated bioinformatics tools and databases. The STRING tool was utilized to analyze the PPI networks of shared DEGs, helping to clarify how these genes interact within the networks (**Figure 5A**). The CytoHubba plugin, using algorithms such as Maximal Clique Centrality, Maximum Neighborhood Component, and Degree Network Centrality Measure, was employed to pinpoint and assess the top 10 hub genes in the PPI network (**Figures 5B-E**). From these, six candidate hub genes were identified at the intersection of these algorithms: *CHD8*, *DCLK1*, *DDX42*, *DHX36*, *EIF3D*, and *GFM1* (**Supplementary Table S7**).

Further analysis using the CHIPBase database (v3.0) allowed for the visualization of interaction networks between the candidate hub genes and various TFs. This revealed 61 interactions involving the 6 hub genes and 33 TFs, which were then visualized using Cytoscape, where mRNAs were represented as blue circles and TFs as red circles (**Figure 5F**, **Supplementary Table S8**). Predictions from the miRDB database, with a Target Score of ≥95, identified 42 target miRNAs for the hub genes. A co-expression network comprising these hub genes and miRNAs was constructed, uncovering 44 mRNA–miRNA pairs (**Figure 5G**, **Supplementary Table S9**).

The mRNA–drug interaction network illustrated potential therapeutic implications by revealing nine potential drugs associated with four of the hub genes: *DCLK1*, *DHX36*, *EIF3D*, and *GFM1* (**Supplementary Figure S3A**, **Supplementary Table S10**). Additionally, the mRNA–RBP interaction network showcased 31 RBPs interacting with 6 hub genes, with *CHD8* specifically targeting 17 RBPs (**Supplementary Figure S3B**, **Supplementary Table S11**). Furthermore, Friends analysis highlighted that DEAH-Box Helicase 36 (*DHX36*) exhibits the strongest correlation with the other hub genes (**Supplementary Figure S3C**), underscoring its potential significance in these diseases.

### Enrichment analysis of hub genes

**Figure 6:**
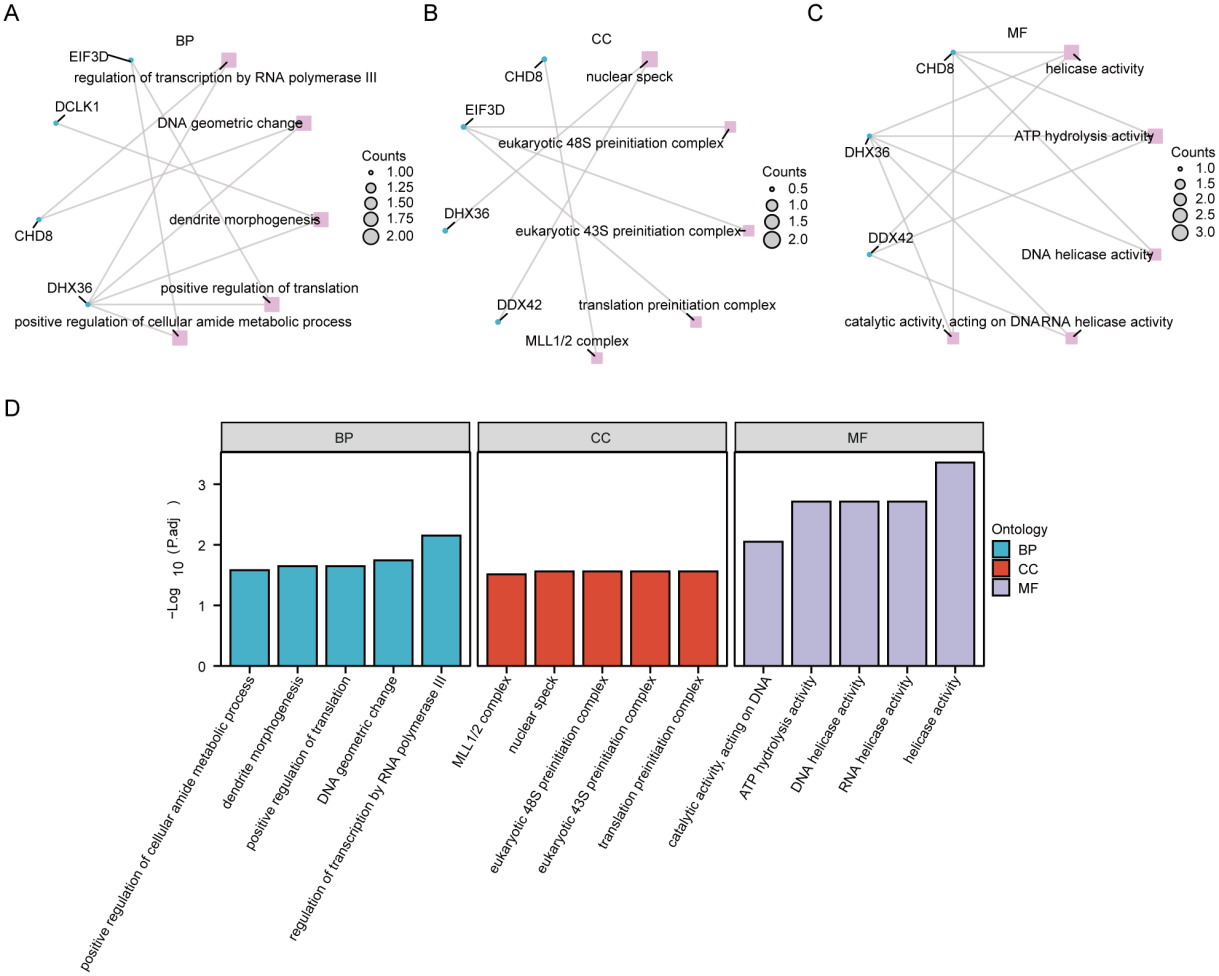
Functional enrichment analysis of hub genes. **(A)** The enrichment in biological process (BP) categories. **(B)** The cellular component (CC) category, illustrating the parts of the cell or extracellular environment where these hub genes are predominantly involved. **(C)** Enrichment in molecular function (MF) categories. **(D)** A histogram of the gene ontology (GO) enrichment analysis, visually representing the number of hub genes associated with various GO terms.

We conducted an enrichment analysis of co-DEGs to understand the biological roles they play in the context of Kyoto Encyclopedia of Genes and Genomes (KEGG) pathways and GO terms. The analysis, depicted in **Figure 6** (**Supplementary Table S12**), reveals that the BP terms identified hub genes predominantly involved in processes such as the regulation of transcription by RNA polymerase III, changes in DNA geometry, dendrite morphogenesis, enhancement of translation, and augmentation of the cellular amide metabolic process. These findings suggest that mechanisms related to gene regulation, cellular shape, and metabolism are crucial in the development of PAH alongside MDD. Concerning CC terms, our findings point to a significant association of hub genes with structures like the nuclear speck, eukaryotic 48S and 43S preinitiation complexes, translation preinitiation complex, and the MLL1/2 complex, highlighting their nuclear involvement. In the realm of MF, the hub genes showed enrichment in functions including helicase activity, ATP hydrolysis, DNA helicase activity, RNA helicase activity, and catalytic activity impacting DNA. This indicates their key role in DNA and RNA processing. Notably, the KEGG pathway analysis did not reveal any statistically significant enrichment.

### Internal cross-dataset validation of hub genes

**Figure 7:**
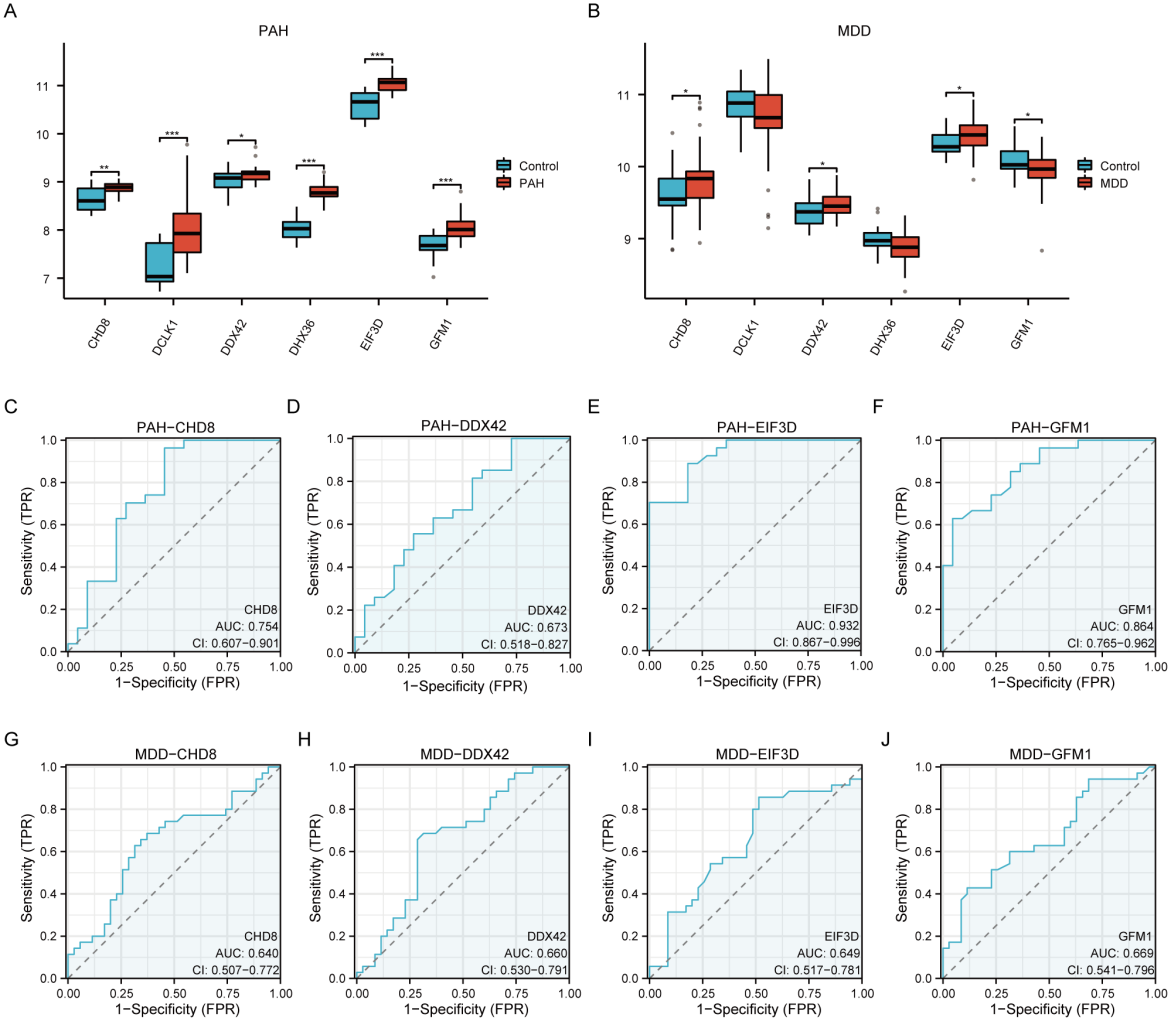
A comprehensive validation of six hub genes within the context of PAH and MDD. **(A, B)** The expression levels of the six hub genes in the PAH **(A)** and MDD **(B)** datasets. The graphical representations highlight differences in gene expression between the disease and control groups, with statistical significance marked by various symbols (ns = not significant, * p < 0.05, ** p < 0.01, *** p < 0.001, **** p < 0.0001), indicating p-values from nonsignificant to highly significant differences. **(C–F)** Receiver operating characteristic (ROC) analysis of the four hub genes (*CHD8*, *DDX42*, *EIF3D*, and *GFM1*) in the PAH dataset. Each panel depicts the ROC curve for one gene, providing the area under the curve (AUC) values: **(C)** ROC curve for *CHD8*. **(D)** ROC curve for *DDX42*. **(E)** ROC curve for *EIF3D*. **(F)** ROC curve for *GFM1*. These curves evaluate the diagnostic effectiveness of each gene, with AUC values assessing their performance as biomarkers. A higher AUC value (closer to 1) indicates a more effective diagnostic outcome. **(G–J)** Similar to panels **(C–F)** but for the MDD dataset, showing the ROC analysis for the same hub genes. **(G)** ROC curve for *CHD8*. **(H)** ROC curve for *DDX42*. **(I)** ROC curve for *EIF3D*. **(J)** ROC curve for *GFM1*. Each panel details the AUC value, reflecting the diagnostic accuracy for MDD, with the same significance markers used to indicate statistical relevance.

We evaluated the differential expression of six pivotal hub genes (*CHD8*, *DCLK1*, *DDX42*, *DHX36*, *EIF3D*, and *GFM1)* within the PAH (GSE113439, GSE53408) and MDD (GSE44593, GSE54564) datasets, as shown in **Figure 7**. Our analysis revealed notable upregulation of all six genes in the PAH cohort compared to the controls (**Figure 7A**). For the MDD cohort, the expression levels of CHD8, DDX42, and EIF3D were elevated, among which, GFM1 expression decreased, while no significant changes in DCLK1 and DHX36 (**Figure 7B**). Further analysis identified that CHD8, DDX42, EIF3D, and GFM1 are differentially expressed across both datasets. We then conducted ROC analysis for these genes. EIF3D demonstrated the highest diagnostic accuracy for PAH, with an AUC of 0.932 (**Figure 7E**). CHD8 and GFM1 displayed moderate diagnostic performance, with AUCs of 0.754 (**Figure 7C**) and 0.864 (**Figure 7F**), respectively, whereas DDX42 showed relatively lower accuracy with an AUC of 0.673 (**Figure 7D**). In the MDD cohorts, the predictive values of these genes were more limited: CHD8 (AUC=0.640; **Figure 7G**), DDX42 (AUC=0.660; **Figure 7H**), EIF3D (AUC=0.649; **Figure 7I**), and GFM1 (AUC=0.669; **Figure 7J**).

Following this, we performed Pearson correlation analysis to compute the correlation coefficients among the key genes. Heatmaps visualizing these correlations are displayed in **Supplementary Figures S4A, C**. Of all of the pairwise correlations evaluated, *DDX42* and *CHD8* showed the strongest positive correlation. To better illustrate their relationship, scatter plots were created. These analyses revealed that in the PAH dataset, the correlation coefficient between *DDX42* and *CHD8* was significant, with R=0.754 (**Supplementary Figure S4B**). In contrast, within the MDD dataset, the correlation was somewhat lower, with R=0.636(**Supplementary Figure S4D**). These findings underscore a robust inter-gene connection, particularly in the PAH context, suggesting potential pathways for further investigation.

### Subtype construction

To delineate disease subtypes within the PAH and MDD datasets, we applied consistency clustering analysis. Analysis of the cumulative distribution function curves of the consensus score matrix and the proportion of ambiguous clustering statistic indicated that the ideal cluster number is two (k=2), as shown in **Supplementary Figures S5B, C, F, G**. Specifically, in the PAH dataset, cluster 1 included 27 samples, and cluster 2 comprised 22 samples, as detailed in **Supplementary Figures S5A–C**. In the MDD dataset, cluster 1 contained 30 samples, while cluster 2 had 40 samples, as illustrated in **Supplementary Figures S5E–G**. Additionally, box plots revealed statistically significant differences in the expression of hub genes between the clusters in the PAH dataset (*p*<0.05), signifying distinct subtypes with potentially varying pathological characteristics. However, in the MDD dataset, the differences in hub gene expression between clusters were not statistically significant, as depicted in **Supplementary Figures S5D, H**.

### Immune cell infiltration analysis

**Figure 8:**
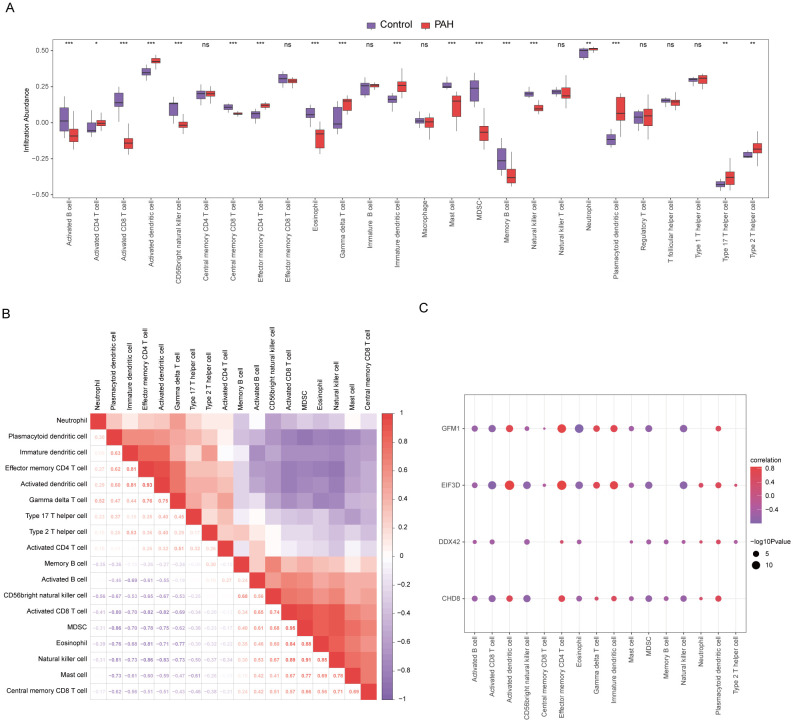
Differences in immune characteristics between the disease and control groups in the PAH dataset through single-sample gene-set enrichment analysis (ssGSEA). **(A)** Subgroup comparison plot of ssGSEA immune infiltration analysis results for PAH versus control in the PAH dataset. **(B)** Correlation analysis of immune cell infiltration abundance differences between the PAH and control groups in the PAH dataset. **(C)** Point plots showing correlations between the infiltration abundances of different immune cells and four key genes in the PAH dataset. Statistical significance indicators (ns = not significant, * p < 0.05, ** p < 0.01, *** p < 0.001, **** p < 0.0001) are used throughout the figure to denote the reliability of the observed differences and correlations, ensuring that readers can quickly discern the most scientifically relevant findings.

**Figure 9:**
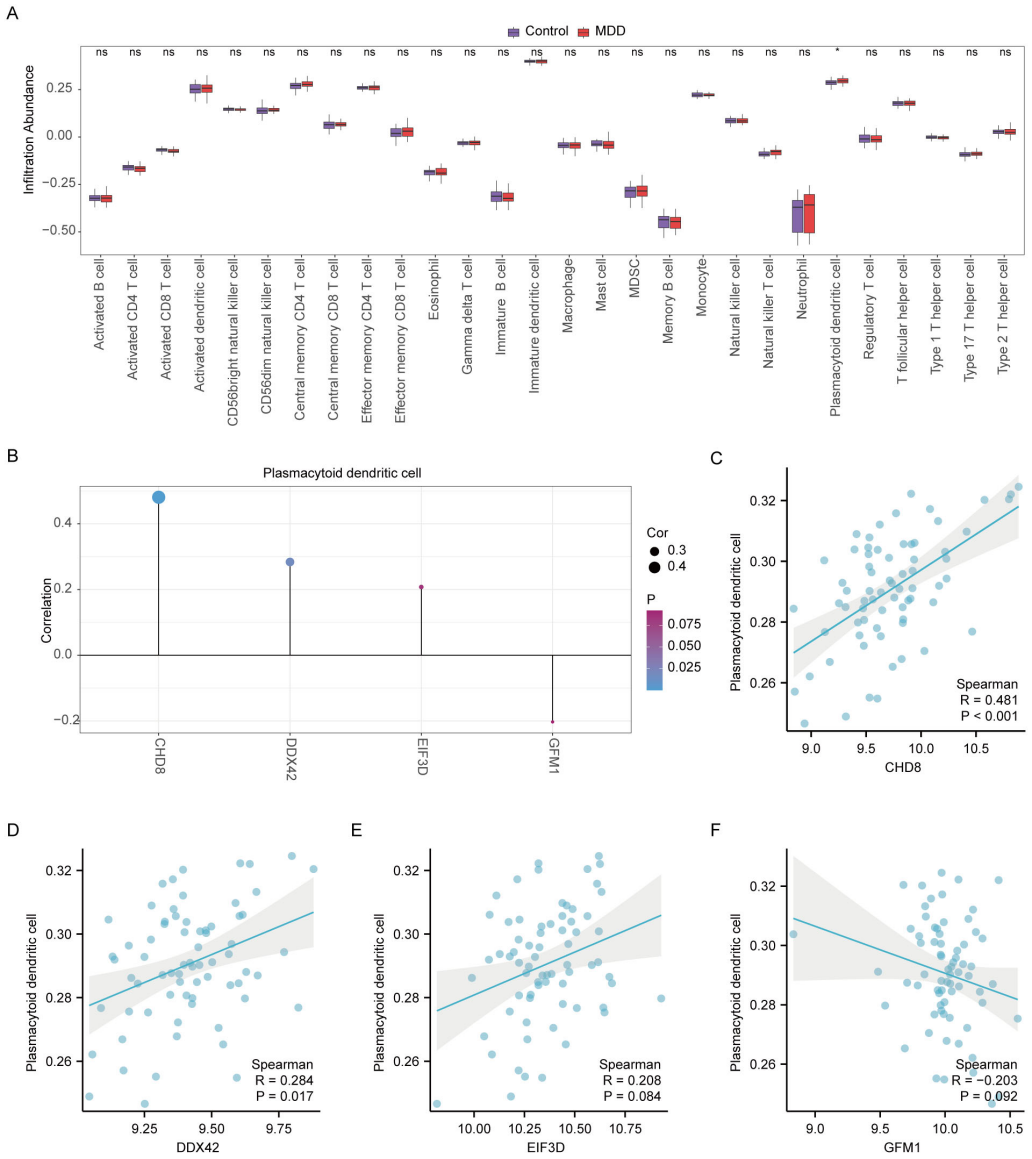
Differential analysis of immune characteristics by single-sample gene-set enrichment analysis (ssGSEA) between the MDD and control groups. **(A)** Subgroup comparative graphical presentation of the ssGSEA immune infiltration analysis results between the MDD and control groups. It visually summarizes the differences in immune cell infiltration, highlighting which types of immune cells are more or less abundant in MDD patients compared to healthy controls. **(B)** Lollipop plot linking the infiltration abundance of plasmacytoid dendritic cells with the expression of four key genes in the MDD dataset. **(C–F)** Scatter plots illustrating the correlation between the expression of each of the four key genes: *CHD8***(C)**, *DDX42***(D)**, *EIF3D***(E)**, and *GFM1***(F)** in the MDD dataset and the infiltration abundance of plasmacytoid dendritic cells. Each scatter plot explores the nature of these correlations, providing insights into how the activity of specific genes may be influenced by or influence the presence of particular immune cells in the context of MDD. Statistical significance indicators (ns = not significant, * p < 0.05, ** p < 0.01, *** p < 0.001, **** p < 0.0001) are used across all panels to denote the levels of statistical significance of the findings.

In our study, the microenvironment, comprising immune cells, the extracellular matrix, inflammatory mediators, and growth factors, was analyzed for its impact on therapeutic sensitivity and diagnostic accuracy. Using the ssGSEA algorithm, we quantified the abundance of 28 immune cell types and identified statistically significant differences in 18 immune cell populations within the PAH dataset (**Figure 8A**), whereas only plasmacytoid dendritic cells showed significant variation in the MDD dataset (**Figure 9A**). Notably, significant positive correlations were found between the hub genes *CHD8*, *DDX42*, and *EIF3D* and plasmacytoid dendritic cells in the PAH dataset (**Figures 8B, C**), suggesting a vital role for these cells in the pathogenesis of PAH in the context of MDD. In the MDD dataset, *CHD8* and *DDX42* also demonstrated positive correlations with plasmacytoid dendritic cells (**Figures 9B–F**), reinforcing the potential importance of these cells.

Additionally, the CIBERSORT algorithm was employed to estimate the proportions of 22 different immune cells in both PAH and MDD (**Supplementary Figures S6A**, **S7A**). In the PAH dataset, immune cell correlations among the 22 immune cell types showed that M2 macrophages, CD8^+^ T cells, resting dendritic cells, and resting mast cells were predominantly positively correlated, while negative correlations with activated natural killer cells, M0 macrophages, eosinophils, activated dendritic cells, plasma cells, and regulatory T cells were observed (**Supplementary Figures S6B, C**). Notably, resting dendritic cells and CD8^+^ T cells were significantly negatively correlated with the four key genes *CHD8*, *DDX42*, *EIF3D*, and *GFM1* (**Supplementary Figure S6D**). Conversely, in the MDD dataset, immune cells generally displayed negative correlations with one another (**Supplementary Figure S7B**). However, *EIF3D* exhibited a significant positive correlation with the abundance of resting memory CD4^+^ T cells, and *DDX42* and *CHD8* correlated positively with the abundance of resting natural killer cells (**Supplementary Figure S7C**).

### Diagnostic model construction

**Figure 10:**
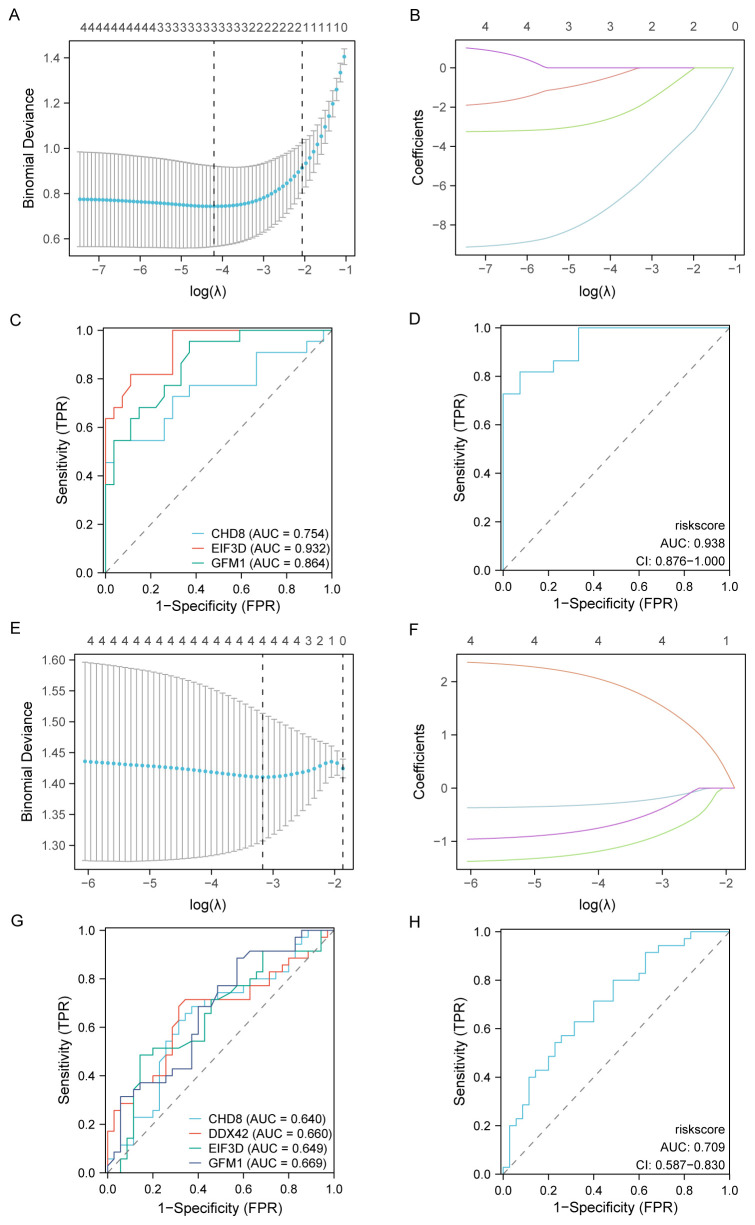
Development and validation of diagnostic models based on immune-related differentially expressed genes (ILRDEGs) for PAH and MDD using LASSO regression analysis. **(A)** The results of ten-fold cross-validation for tuning parameter (λ) selection in the LASSO regression model for PAH. This plot helps to determine the optimal λ value that minimizes prediction error, which is crucial for enhancing the model’s accuracy. **(B)** The coefficient profiles of variables in the LASSO regression model for PAH. This graph traces the paths of coefficients as λ changes, illustrating how the inclusion of each variable in the model is affected by regularization, which helps to select the most significant predictors. **(C)** Receiver operating characteristic (ROC) curves of hub genes in PAH. This panel assesses the diagnostic performance of individual genes, providing a clear comparison of their ability to discriminate between disease and control states. **(D)** The ROC curve of the risk score in PAH, computed based on the LASSO model. This curve evaluates the overall diagnostic accuracy of the combined model, showing the effectiveness of the risk score in predicting PAH. **(E)** Ten-fold cross-validation for λ selection in the LASSO model for the MDD dataset. This panel aids in identifying the best regularization parameter to prevent overfitting while maintaining model performance. **(F)** The coefficient profiles of variables in the LASSO regression model for MDD. This panel highlights how variables are selected and their coefficients shrink as λ increases, focusing on the most impactful predictors. **(G)** ROC curves for hub genes in MDD, which can be used to analyze each gene’s diagnostic power and its utility as a biomarker for detecting MDD. **(H)** The ROC curve of the risk score in MDD, generated by the LASSO model. This panel assesses the predictive performance of the risk score, indicating its potential as a diagnostic tool for MDD.

For assessing the diagnostic potential of hub genes, we utilized LASSO logistic regression within our study (**Figures 10A, E**). Furthermore, we visualized the results of the LASSO regression and generated the corresponding LASSO variable coefficient path plots (**Figures 10B, F**). In the PAH dataset, three genes were identified as potential biomarkers: *CHD8*, *EIF3D*, and *GFM1.* The evaluation of these genes showed promising results, with AUC values of 0.754 for *CHD8*, 0.932 for *EIF3D*, and 0.864 for *GFM1*, indicating their significant diagnostic value (**Figures 10C, D**).

In contrast, within the MDD dataset, four genes were highlighted as potential biomarkers: *CHD8*, *DDX42*, *EIF3D*, and *GFM1.* Their diagnostic effectiveness was assessed, revealing AUC values of 0.640 for *CHD8*, 0.660 for *DDX42*, 0.649 for *EIF3D*, and 0.669 for *GFM*1, as illustrated in **Figures 10G, H**. These values suggest the moderate diagnostic utility of these biomarkers for MDD, highlighting the need for further validation and potentially the exploration of additional markers to improve diagnostic accuracy.

### *In-vivo* model and further validation of the hub genes

**Figure 11:**
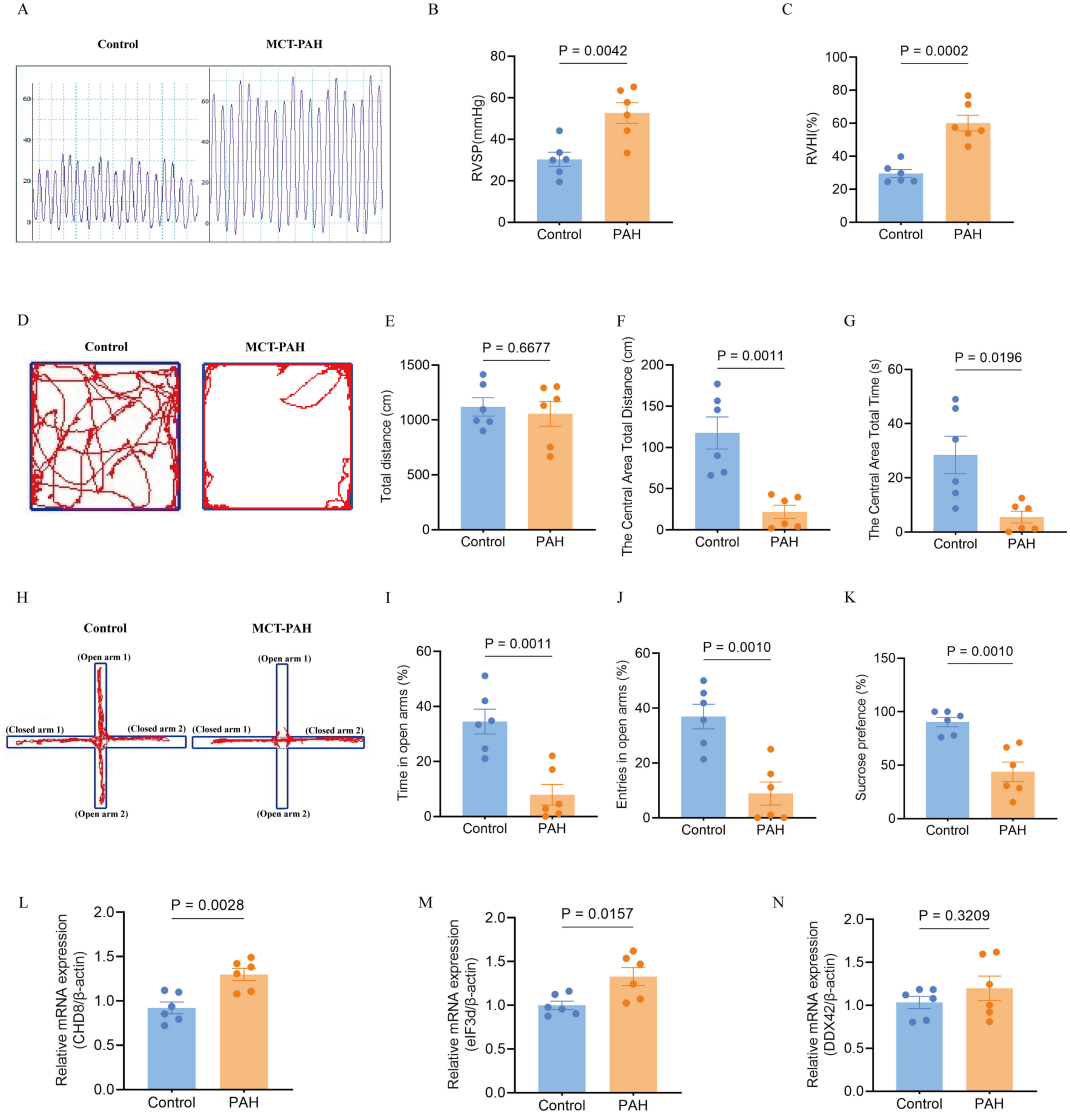
Effects of monocrotaline (MCT)-induced PAH on physiological parameters, behavioral outcomes, and hub gene expression in the rat model. **(A–C)**. Characterization of PAH, showing elevated right ventricular systolic pressure (RVSP) **(A, B)** and right ventricular hypertrophy index (RVHI) **(C)**. These parameters indicate the severity of PAH in the model (n = 6 per group; independent-samples t-test). **(D–G)** Open field test (OFT) for anxiety-like behavior. **(D)** Representative movement traces; **(E)** total distance traveled; **(F)** distance traveled in the central area; **(G)** time spent in the central area (reduced values indicate higher anxiety). (n = 6; independent-samples t-test for **(E, F)**, Mann–Whitney U test for **G**). **(H–J)** Elevated plus maze (EPM) for anxiety-like behavior. **(H)** Representative movement traces; **(I)** time spent in open arms; **(J)** number of open-arm entries (greater exploration indicates lower anxiety). (n = 6; independent-samples t-test). **(K)** Sucrose preference test (SPT) for depression-like behavior, with reduced sucrose preference indicating a depressive-like state (n = 6; independent-samples t-test). **(L–N)** Expression of hub genes in the PAH model: relative mRNA levels of CHD8 **(L)**, EIF3D **(M)**, and DDX42 **(N)**. (n = 6; independent-samples t-test). Data are shown as mean ± SEM. Statistical significance was assessed using independent-samples t-tests or Mann–Whitney U tests, as indicated.

In line with prior research, our *in-vivo* model utilizing monocrotaline to induce PAH in rats revealed typical symptoms such as increased right ventricular systolic pressure and right ventricular hypertrophy index, as depicted in **Figures 11A–C**. For the first time, our study also identified negative emotional behaviors in PAH rats, as evidenced by their performance in behavioral tests. In the open field test, the PAH rats showed a reduced distance and time spent in the center compared to the control rats, indicating increased anxiety (**Figures 11D, F, G**), while the overall distance traveled remained unchanged (**Figure 11E**). Similarly, in the elevated-plus-maze test, the PAH rats exhibited a decrease in retention time and fewer entries into the open arms relative to the controls, further supporting signs of anxiety and depression (**Figures 11H–J**). The sucrose preference test underscored these findings, with the PAH rats showing a decreased preference for the sucrose solution, suggesting anxiety- and depressive-like behaviors (**Figure 11K**).

To further validate the involvement of hub genes, we conducted quantitative qPCR analysis on lung tissues from both the PAH and control groups. Our results confirmed significant upregulation of *CHD8* and *EIF3D* in the PAH group compared to the controls (**Figures 11L, M**). *DDX42* also showed an elevation in the expression levels in the PAH group, although the difference did not reach statistical significance (**Figure 11N**). These transcriptional changes align with our earlier bioinformatics findings, reinforcing the role of these genes in the pathogenesis of PAH and its associated emotional disturbances.

## Discussion

To identify common differentially expressed genes (Co-DEGs) associated with both pulmonary arterial hypertension (PAH) and major depressive disorder (MDD), a comprehensive analysis of multiple datasets was performed in this research, using the combination of bioinformatics tools. The overlapping pathogenic genes between these conditions are identified for the first time. Through analyses such as protein-protein interaction, pathway enrichment, and immune infiltration studies, the potential pathogenic mechanisms underlying PAH-associated MDD were explored, which are related to inflammatory and immune processes. These include the IL-12 signaling pathway, the Notch signaling pathway, the interferon signaling pathway, the neuroactive ligand-receptor interaction pathway, and plasmacytoid dendritic cell immune infiltration. Moreover, three pivotal co-hub genes (*CHD8*, *DDX42*, and *EIF3D*) were identified by using machine learning techniques, which were later validated in a PAH rat model. This study is the first to report that PAH rats, which exhibit elevated expression of these genes, display anxiety and depression-like behaviors.

Depression is a common complication associated with PAH. The impact of depression on the health and quality of life has been reported in literatures (36–38). However, except for reports on its prevalence, there is rare publication of the large-scale, high-quality, prospective population-based cohort studies, with the focus on defining the diagnostic criteria and specific mechanisms underlying PAH-related major depressive disorder (MDD). Investigating objective predictive biomarkers from a biological perspective could facilitate earlier and more effective interventions or preventive measures in these patients.

It has been reported that compared to the general population, patients with pulmonary hypertension (PH) have a higher incidence of depression (39, 40), but the MDD incidence is different across different PH subgroups. Up to 53% of PAH patients experienced depression (41). Although it is a comorbidity for the PH patients, their treatment efficacy is also affected by depression, which could cause worse clinical outcomes, such as a reduced exercise capacity measured by the 6-minute walk distance (6MWD), an important prognostic indicator in PH. Additionally, the PAH patients with depression may have poorer hemodynamic profiles and higher hospitalization rates (40, 42). Therefore, it is essential to develop novel associative findings for the PAH patients with depression, offering a more potential measure of their disease status.

PAH is marked by increased pulmonary vascular resistance due to lung remodeling or vasoconstriction, leading to severe cardiopulmonary issues and premature mortality (43, 44). Either depression, or MDD is associated with neurotransmitter imbalance, neuroendocrine dysregulation, and immune inflammation (42).

*CHD8* is pivotal in neural development and is implicated in both MDD and PAH. As an ATP-dependent chromatin remodeling factor, *CHD8* influences neuronal differentiation, cell cycle progression, and proliferation, which are critical for brain development and function (45–48). It is also linked to autism and intellectual disability (49), with research showing that *CHD8* mutations in mice lead to anxiety and depression-like symptoms. Additionally, the proliferation of vascular smooth muscle cells is recognized as a pathological hallmark of pulmonary hypertension (50, 51). Furthermore, disruption of a single copy of *CHD8* in human neural precursor cells has been shown to alter the cell cycle, potentially impacting cell proliferation—a key factor in PAH pathology (52, 53).

*EIF3D* is crucial for initiating protein synthesis and affects the translation of specific mRNA molecules, influencing cellular phenotype transitions from proliferation to migration (54). This action facilitated through the modulation of *EIF3D*-mediated mRNA translation, is vital in the vascular proliferation found in PAH (55). Additionally, *EIF3D* plays a significant role in the cellular response to sustained endoplasmic reticulum stress, a known pathogenic factor in depression, by regulating the expression of the m6A demethylase ALKBH5 (56, 57).

*DDX42* participates in key RNA processes such as translation initiation, splicing, and ribosome biogenesis (58). Its role in modulating mRNA splice isoforms and vascular smooth muscle cell function could be instrumental in PAH. *DDX42* is also crucial for the regulation of neurotransmitter mRNA splicing and translation, affecting neurotransmitter dynamics and potentially providing a therapeutic target for MDD, given its role in neurotransmitter balance.

In the literature, the lung-brain axis refers to a two-way communication channel between the lungs and the brain, including the complex interactions between the nervous, endocrine, and immune systems (59–61). Within this pathway, the vagus nerve, the immune and neuroendocrine systems and various neurotransmitters act as essential links, each with distinct functions (61, 62). Neuroinflammation and altered autonomic functions have been found to be associated with the systemic manifestation of PAH, supporting extension of the disease beyond the pulmonary vasculature (63) and existence of the lung-brain axis, where immune dysregulation and inflammation are key factors (64).

Immune and inflammatory processes are important in both PAH and MDD, raising the possibility that patients with both conditions may show overlapping immune changes. In our analysis, plasmacytoid dendritic cells (pDCs) stood out, as their infiltration correlated with the hub genes we identified. pDCs produce large amounts of type I interferons and act as regulators of immune activity. In PAH, pDCs have been reported to accumulate around pulmonary vessels, where they release interferon-induced chemokines such as CXCL10 and contribute to vascular remodeling (65). The correlation we observed between pDC infiltration and hub gene expression suggests that these genes may be involved in pDC recruitment or activation. This fits with clinical observations: patients receiving long-term interferon-α treatment, which reproduces a high interferon state, often develop depressive symptoms or MDD (66), and altered pDC function has also been described in depression (67).

There is also evidence that neuronal pathways can influence pDCs. For example, intestinal neurons can regulate their activity through serotonin–HTR7 signaling (68). Serotonin plays a recognized role in both pulmonary hypertension and depression: signaling through receptors such as 5-HT1B promotes vascular remodeling in PAH (69), while changes in serotonin binding and metabolism are linked to mood disturbances in MDD (70). Taken together, these findings suggest that pDCs may act as a connecting element within the lung–brain axis, linking immune activation, vascular remodeling, and neurotransmitter pathways in PAH with depression.

### Limitations and outlook of the study

This study has several limitations. The bioinformatics analysis was based on GEO datasets rather than direct sequencing, which may influence the reliability of the results. For molecular validation, qPCR was carried out only on lung tissue from MCT-induced PAH rats. These findings mainly reflect pulmonary changes in PAH and do not establish a direct role in MDD. Thus, we cannot confirm their causal involvement in the comorbidity mechanism of PAH–MDD. Extrapolation from lung tissue to central nervous system pathology should therefore be cautious. In addition, evidence at the protein level and functional confirmation of hub genes is still lacking. To clarify causality, future work will need *in vitro* and *in vivo* experiments in which these genes are silenced or overexpressed in relevant cells and animal models, with effects measured on vascular remodeling and behavior. Validation at the protein level, such as Western blotting, ELISA, and immunohistochemistry in both rat and patient samples, will also be required. Candidate biomarkers including CHD8, DDX42, and EIF3D should be assessed in larger, independent cohorts of patients with PAH, with and without depression, as well as in MDD cohorts, to determine their diagnostic or prognostic potential.

The immune infiltration analysis was performed using ssGSEA (Bindea 28-cell signatures) and CIBERSORT (LM22). These tools were originally developed for blood samples, and their accuracy in solid tissues such as lung and brain may be limited. Tissue heterogeneity, sampling variation, and the local microenvironment can all influence the estimates, meaning the results reflect local rather than systemic immune activity. Future studies combining blood profiling with single-cell or spatial transcriptomic approaches will help to resolve these issues and provide more reliable cell-type–specific information.

In conclusion, the present results are based on transcriptomic correlations and cannot be taken as evidence of causality or immediate diagnostic utility. Additional functional experiments, protein-level studies, and validation in clinical cohorts are necessary to confirm the biological and clinical relevance of the identified genes.

## Conclusion

This research highlights the genetic and molecular links between PAH and MDD by examining gene expression data. It identified 42 DEGs common to both conditions. Through PPI network analysis and LASSO regression modeling, crucial genes such as *CHD8*, *DDX42*, *EIF3D*, and *GFM1* were pinpointed. Notably, *CHD8*, *DDX42*, and *EIF3D* emerged as predictive markers to the overlapping pathophysiological processes of both diseases. These findings provide novel insights into potential therapeutic targets and suggest directions for future investigations.

## Data Availability

Publicly available datasets were analyzed in this study. This data can be found here: The PAH datasets GSE113439 and GSE53408 as well as the MDD datasets GSE44593 and GSE54564 were retrieved from the Gene Expression Omnibus (GEO) database at https://www.ncbi.nlm.nih.gov/geo/.

## Glossary

AUC: area under the curve
BP: biological process
CC: cellular component
*CHD8*: chromatin helicase DNA-binding protein 8
Co-DEGs: common differentially expressed genes
*DDX42*: DEAD-box helicase 42
DEGs: differentially expressed genes
*DHX36*: DEAH-Box Helicase 36
*EIF3D*: eukaryotic translation initiation factor 3, subunit D
GEO: Gene Expression Omnibus
GO: Gene Ontology
GSEA: Gene Set Enrichment Analysis
GSVA: Gene Set Variation analysis
HPA: hypothalamic–pituitary–adrenal
IL: interleukin
KEGG: Kyoto Encyclopedia of Genes and Genomes
Logistic-LASSO: Logistic-Least Absolute Shrinkage and Selection Operator
MDD: major depressive disorder
MF: molecular function
PAH: Pulmonary arterial hypertension
PPI: protein–protein interaction
PID: primary immunodeficiency
PPI: Protein–Protein Interaction
qPCR: quantitative polymerase chain reaction
RBP: RNA-binding protein
ROC: receiver operating characteristic
RVHI: right ventricular hypertrophy index
ssGSEA: single-sample gene-set enrichment analysis;TF, transcription factor
