# Supplementary material for: Identification and experimental validation of common genes associated with both pulmonary arterial hypertension and major depressive disorder
Source: Front Psychiatry. 2025 Nov 7;16:1670519. doi: 10.3389/fpsyt.2025.1670519 (PMC12634556; doi:10.3389/fpsyt.2025.1670519)
Supplement: Supplementary file 1 [file Table1.docx]

**Table S1. List of GEO dataset information**

|  | GSE113439 | GSE53408 | GSE44593 | GSE54564 |
| --- | --- | --- | --- | --- |
| Platform | GPL6244 | GPL6244 | GPL570 | GPL6947 |
| Species | *Homo sapiens* | *Homo sapiens* | *Homo sapiens* | *Homo sapiens* |
| Tissue | Fresh frozen lung | Fresh frozen lung | brain_AMY | brain amygdala |
| samples in the disease group | PAH: 15 | PAH: 12 | MDD: 14 | MDD: 21 |
| Samples in the control group | 11 | 11 | 14 | 21 |
| Reference | Osteopontin lung gene expression is a marker of disease severity in pulmonary arterial hypertension | A biochemical approach to understand the pathogenesis of advanced pulmonary arterial hypertension: Metabolomic profiles of arginine, sphingosine-1-phosphate, and heme of human lung |  | A conserved BDNF-, glutamate-, and GABA-enriched gene module related to human depression identified by coexpression meta-analysis and DNA variant genome-wide association studies |

BNDF, brain-derived neurotrophic factor; GABA, gamma-aminobutyric acid; MDD, major depressive disorder; PAH, pulmonary arterial hypertension.

**Table S2. Common differentially expressed genes in the PAH dataset and MDD dataset**

| Common differentially expressed genes | |
| --- | --- |
| *ADAMTS3* | *ECD* |
| *AIFM1* | *EFNB1* |
| *AP3B1* | *EIF3D* |
| *ATHL1* | *ERO1L* |
| *ATIC* | *FADS3* |
| *ATP6V1A* | *FAM111A* |
| *BECN1* | *FBXO28* |
| *C12orf5* | *FEM1C* |
| *C9orf116* | *FGL2* |
| *CADPS2* | *GFM1* |
| *CCDC25* | *GFPT1* |
| *CD52* | *GPR124* |
| *CDK5RAP2* | *H1FX* |
| *CFB* | *HELZ* |
| *CHD8* | *HMCN1* |
| *CHD9* | *HMGCS1* |
| *DBF4* | *HYAL2* |
| *DCLK1* | *ICAM2* |
| *DDX42* | *ITSN1* |
| *DHX36* | *KANK2* |
| *DOCK6* | *KIAA1462* |

PAH, pulmonary arterial hypertension; MDD, major depressive disorder.

**Table S3. Gene set enrichment analysis results of the PAH dataset**

| ID | Set size | Enrichment score | NES | *p*-value | Adjusted *p*-value | q value |
| --- | --- | --- | --- | --- | --- | --- |
| REACTOME_SIGNALING_BY_NOTCH2 | 10 | -0.63602 | -1.61498 | 0.030075 | 0.171384 | 0.14153 |
| REACTOME_SIGNALING_BY_NOTCH4 | 20 | -0.52912 | -1.60529 | 0.029006 | 0.170105 | 0.140474 |
| REACTOME_TP53_REGULATES_TRANSCRIPTION_OF_DNA_REPAIR_GENES | 10 | 0.632399 | 1.807927 | 0.023739 | 0.1508 | 0.124532 |
| WP_NOTCH_SIGNALING | 10 | -0.65991 | -1.67563 | 0.018045 | 0.130471 | 0.107744 |
| REACTOME_REGULATION_OF_TP53_ACTIVITY_THROUGH_PHOSPHORYLATION | 22 | 0.467018 | 1.750539 | 0.008065 | 0.089183 | 0.073648 |
| PID_IL12_2PATHWAY | 15 | -0.62113 | -1.75372 | 0.007153 | 0.083994 | 0.069363 |

PAH, pulmonary arterial hypertension.

**Table S4. Gene set enrichment analysis results of the MDD dataset**

| ID | Set size | Enrichment score | NES | *p*-value | Adjusted *p-*value | q value |
| --- | --- | --- | --- | --- | --- | --- |
| KEGG_OXIDATIVE_PHOSPHORYLATION | 47 | -0.64268 | -2.23197 | 0.002033 | 0.152686 | 0.141942 |
| KEGG_GNRH_SIGNALING_PATHWAY | 40 | 0.575487 | 1.946916 | 0.001923 | 0.152686 | 0.141942 |
| REACTOME_LAMININ_INTERACTIONS | 28 | 0.616617 | 1.915196 | 0.002075 | 0.152686 | 0.141942 |
| REACTOME_INTERFERON_SIGNALING | 93 | 0.419761 | 1.681176 | 0.004115 | 0.159956 | 0.148702 |
| REACTOME_ION_CHANNEL_TRANSPORT | 92 | -0.41234 | -1.62419 | 0.003899 | 0.159956 | 0.148702 |
| KEGG_NEUROACTIVE_LIGAND_RECEPTOR_INTERACTION | 159 | -0.35471 | -1.51994 | 0.003914 | 0.159956 | 0.148702 |

MDD, major depressive disorder.

**Table S5. Gene set variation analysis of the PAH dataset**

| ID | logFC | AveExpr | t | *p*-value | Adjusted *p*-value | B |
| --- | --- | --- | --- | --- | --- | --- |
| REACTOME_WNT5A_DEPENDENT_INTERNALIZATION_OF_FZD4 | 1.351623243 | 7.31E-05 | 16.55501506 | 2.51E-22 | 5.92E-21 | 40.35572691 |
| REACTOME_WNT5A_DEPENDENT_INTERNALIZATION_OF_FZD2_FZD5_AND_ROR2 | 1.351623243 | 7.31E-05 | 16.55501506 | 2.51E-22 | 5.92E-21 | 40.35572691 |
| REACTOME_SIGNALING_BY_MEMBRANE_TETHERED_FUSIONS_OF_PDGFRA_OR_PDGFRB | 1.340241662 | -0.005432915 | 16.35251718 | 4.29E-22 | 9.79E-21 | 39.81403529 |
| REACTOME_PHASE_3_RAPID_REPOLARISATION | 1.33935437 | 0.000804335 | 13.66970935 | 8.24E-19 | 1.14E-17 | 32.18313766 |
| REACTOME_EFFECTS_OF_PIP2_HYDROLYSIS | 1.325161323 | 0.004416528 | 15.97359652 | 1.19E-21 | 2.54E-20 | 38.7879489 |
| REACTOME_SCAVENGING_BY_CLASS_B_RECEPTORS | -1.607698265 | -0.062204316 | -32.46385274 | 4.36E-36 | 8.67E-34 | 72.18863573 |
| WP_EFFECT_OF_INTESTINAL_MICROBIOME_ON_ANTICOAGULANT_RESPONSE_OF_VITAMIN_K_ANTAGONISTS | -1.607698265 | -0.062204316 | -32.46385274 | 4.36E-36 | 8.67E-34 | 72.18863573 |
| WP_FAMILIAL_HYPERLIPIDEMIA_TYPE_3 | -1.607698265 | -0.062204316 | -32.46385274 | 4.36E-36 | 8.67E-34 | 72.18863573 |
| WP_METABOLIC_PATHWAY_OF_LDL_HDL_AND_TG_INCLUDING_DISEASES | -1.607698265 | -0.062204316 | -32.46385274 | 4.36E-36 | 8.67E-34 | 72.18863573 |
| PID_P38_GAMMA_DELTA_PATHWAY | -1.614807456 | -0.063059835 | -36.41591225 | 1.46E-38 | 8.11E-36 | 77.8566081 |

logFC, logarithm of the absolute fold change; PAH, pulmonary arterial hypertension.

**Table S6. Gene set variation analysis of the MDD dataset**

| ID | logFC | AveExpr | t | *p*-value | adj.P.Val | B |
| --- | --- | --- | --- | --- | --- | --- |
| REACTOME_RECEPTOR_TYPE_TYROSINE_PROTEIN_PHOSPHATASES | 0.422332922 | 0.022970775 | 3.178166136 | 0.002160736 | 0.957640578 | -2.264524287 |
| REACTOME_RUNX3_REGULATES_BCL2L11_BIM_TRANSCRIPTION | 0.370168822 | -0.016567805 | 2.496234829 | 0.014769639 | 0.957640578 | -3.27184664 |
| REACTOME_MISMATCH_REPAIR | 0.347936004 | 0.011431417 | 2.429274158 | 0.017546772 | 0.957640578 | -3.36089685 |
| KEGG_MISMATCH_REPAIR | 0.347936004 | 0.011431417 | 2.429274158 | 0.017546772 | 0.957640578 | -3.36089685 |
| WP_DNA_MISMATCH_REPAIR | 0.347936004 | 0.011431417 | 2.429274158 | 0.017546772 | 0.957640578 | -3.36089685 |
| REACTOME_TRANSPORT_OF_NUCLEOSIDES_AND_FREE_PURINE_AND_PYRIMIDINE_BASES_ACROSS_THE_PLASMA_MEMBRANE | -0.304115146 | -0.04072011 | -2.089338729 | 0.040101795 | 0.957640578 | -3.782592852 |
| BIOCARTA_SAM68_PATHWAY | -0.316295534 | 0.027145559 | -2.248955076 | 0.027479687 | 0.957640578 | -3.591052052 |
| WP_DISORDERS_IN_KETOLYSIS | -0.322658799 | 0.049754533 | -2.430081888 | 0.017510661 | 0.957640578 | -3.359834023 |
| REACTOME_MASTL_FACILITATES_MITOTIC_PROGRESSION | -0.334301318 | 0.030735091 | -2.161209774 | 0.033902045 | 0.957640578 | -3.697797553 |

logFC, logarithm of the absolute fold change; MDD, major depressive disorder.

**Table S7. Table of hub genes**

| Hub genes | Full name |
| --- | --- |
| CHD8 | Chromodomain Helicase DNA Binding Protein 8 |
| DCLK1 | Doublecortin Like Kinase 1 |
| DDX42 | DEAD-Box Helicase 42 |
| DHX36 | DEAH-Box Helicase 36 |
| EIF3D | Eukaryotic Translation Initiation Factor 3 Subunit D |
| GFM1 | G Elongation Factor Mitochondrial 1 |

**Table S8. mRNA-TF interaction network nodes.**

| mRNA | Transcription factor | | mRNA | Transcription factor |
| --- | --- | --- | --- | --- |
| CHD8 | CTCF |  | DHX36 | CREB1 |
| CHD8 | E2F1 |  | EIF3D | CTCF |
| CHD8 | ERG |  | EIF3D | E2F1 |
| CHD8 | ETS1 |  | EIF3D | EBF1 |
| CHD8 | FOXA1 |  | EIF3D | ELF1 |
| CHD8 | GABPA |  | EIF3D | EP300 |
| CHD8 | GATA1 |  | EIF3D | ERG |
| CHD8 | SPI1 |  | EIF3D | ETS1 |
| CHD8 | CEBPA |  | EIF3D | ETV1 |
| DCLK1 | TFAP2C |  | EIF3D | FLI1 |
| DDX42 | MAX |  | EIF3D | FOS |
| DDX42 | USF1 |  | EIF3D | FOSL2 |
| DDX42 | CEBPA |  | EIF3D | GABPA |
| DDX42 | CEBPB |  | EIF3D | JUN |
| DDX42 | CREB1 |  | EIF3D | JUND |
| DDX42 | CTCF |  | EIF3D | MAX |
| DDX42 | E2F1 |  | EIF3D | MYC |
| DDX42 | E2F6 |  | EIF3D | NRF1 |
| DDX42 | ELF1 |  | EIF3D | BRD2 |
| DDX42 | EP300 |  | EIF3D | POLR2A |
| DDX42 | ERG |  | EIF3D | SMARCA4 |
| DDX42 | ETV1 |  | EIF3D | SPI1 |
| DDX42 | FOXA1 |  | EIF3D | TBP |
| DDX42 | GABPA |  | EIF3D | YY1 |
| DHX36 | ERG |  | EIF3D | CREB1 |
| DHX36 | ETS1 |  | GFM1 | HNF4A |
| DHX36 | FOXA1 |  | GFM1 | EP300 |
| DHX36 | GABPA |  | GFM1 | ERG |
| DHX36 | MAX |  | GFM1 | ETS1 |
| DHX36 | AR |  | GFM1 | GABPA |
| DHX36 | CEBPB |  |  |  |

TF: Transcription factors.

**Table S9. mRNA-miRNA interaction network nodes.**

| mRNA | miRNA |  | mRNA | miRNA |
| --- | --- | --- | --- | --- |
| DHX36 | hsa-miR-190a-3p | | DCLK1 | hsa-miR-3646 |
| DHX36 | hsa-miR-5011-5p | | DCLK1 | hsa-miR-548z |
| DCLK1 | hsa-miR-4668-5p | | CHD8 | hsa-miR-4640-5p |
| DCLK1 | hsa-miR-15b-5p | | CHD8 | hsa-miR-4726-5p |
| DCLK1 | hsa-miR-497-5p | | DCLK1 | hsa-miR-4282 |
| DCLK1 | hsa-miR-16-5p | | DCLK1 | hsa-miR-498-5p |
| DCLK1 | hsa-miR-6838-5p | | DCLK1 | hsa-miR-4770 |
| DCLK1 | hsa-miR-15a-5p | | DCLK1 | hsa-miR-1245b-3p |
| DCLK1 | hsa-miR-195-5p | | DCLK1 | hsa-miR-570-3p |
| DCLK1 | hsa-miR-424-5p | | DCLK1 | hsa-miR-6088 |
| DCLK1 | hsa-miR-548c-3p | | DCLK1 | hsa-miR-3157-5p |
| DCLK1 | hsa-miR-4775 | | DCLK1 | hsa-miR-143-3p |
| GFM1 | hsa-miR-548g-3p | | DHX36 | hsa-miR-493-5p |
| DCLK1 | hsa-miR-548h-3p | | DHX36 | hsa-miR-3529-3p |
| DCLK1 | hsa-miR-548ac | | DHX36 | hsa-miR-4775 |
| DCLK1 | hsa-miR-4524a-5p | | DDX42 | hsa-miR-8063 |
| DCLK1 | hsa-miR-659-3p | | CHD8 | hsa-miR-2355-5p |
| DCLK1 | hsa-miR-548d-3p | | DCLK1 | hsa-miR-17-3p |
| DCLK1 | hsa-miR-627-3p | | DCLK1 | hsa-miR-498-3p |
| DCLK1 | hsa-miR-4524b-5p | | DCLK1 | hsa-miR-1305 |
| DCLK1 | hsa-miR-5011-5p | | DCLK1 | hsa-miR-607 |
| DCLK1 | hsa-miR-548bb-3p | | DCLK1 | hsa-miR-548e-5p |

**Table S10. mRNA-Drugs interaction network nodes.**

| mRNA | Drugs |
| --- | --- |
| DCLK1 | Estradiol |
| DCLK1 | (+)-JQ1 compound |
| DCLK1 | methylmercuric chloride |
| DCLK1 | Tetrachlorodibenzodioxin |
| DCLK1 | Tobacco Smoke Pollution |
| DCLK1 | trichostatin A |
| DCLK1 | Valproic Acid |
| DHX36 | Acetaminophen |
| DHX36 | trichostatin A |
| DHX36 | Valproic Acid |
| EIF3D | Cyclosporine |
| EIF3D | Valproic Acid |
| GFM1 | Valproic Acid |

**Table S11. mRNA-RBP interaction network nodes.**

| mRNA | RBP |
| --- | --- |
| CHD8 | CPSF7 |
| CHD8 | CSTF2T |
| CHD8 | DDX3X |
| CHD8 | DDX54 |
| CHD8 | ELAVL1 |
| CHD8 | HNRNPA2B1 |
| CHD8 | HNRNPC |
| CHD8 | IGF2BP2 |
| CHD8 | PRPF8 |
| CHD8 | RBM10 |
| CHD8 | RBMX |
| CHD8 | RNPS1 |
| CHD8 | SCAF4 |
| CHD8 | SCAF8 |
| CHD8 | TARDBP |
| CHD8 | U2AF1 |
| CHD8 | U2AF2 |
| DCLK1 | FUS |
| DDX42 | ELAVL1 |
| DDX42 | HNRNPC |
| DDX42 | IGF2BP2 |
| DDX42 | RBMX |
| DDX42 | U2AF1 |
| DHX36 | ELAVL1 |
| DHX36 | HNRNPC |
| DHX36 | RBMX |
| DHX36 | U2AF1 |
| DHX36 | U2AF2 |
| EIF3D | RBMX |
| GFM1 | ELAVL1 |
| GFM1 | RBMX |

**Table S12. GO enrichment analysis results of 6 hub genes.**

| ONTOLOGY | ID | Description | pvalue | p.adjust | qvalue |
| --- | --- | --- | --- | --- | --- |
| BP | GO:0006359 | regulation of transcription by RNA polymerase III | 3.43E-05 | 0.007038 | 0.002963 |
| BP | GO:0032392 | DNA geometric change | 0.000382 | 0.018021 | 0.007588 |
| BP | GO:0048813 | dendrite morphogenesis | 0.000798 | 0.022558 | 0.009498 |
| BP | GO:0045727 | positive regulation of translation | 0.000821 | 0.022558 | 0.009498 |
| BP | GO:0034250 | positive regulation of cellular amide metabolic process | 0.001149 | 0.026179 | 0.011023 |
| CC | GO:0016607 | nuclear speck | 0.006226 | 0.027394 | 0.011797 |
| CC | GO:0033290 | eukaryotic 48S preinitiation complex | 0.004585 | 0.027394 | 0.011797 |
| CC | GO:0016282 | eukaryotic 43S preinitiation complex | 0.005195 | 0.027394 | 0.011797 |
| CC | GO:0070993 | translation preinitiation complex | 0.0055 | 0.027394 | 0.011797 |
| CC | GO:0044665 | MLL1/2 complex | 0.00976 | 0.030675 | 0.013209 |
| MF | GO:0004386 | helicase activity | 1.15E-05 | 0.000437 | 0.000133 |
| MF | GO:0016887 | ATP hydrolysis activity | 0.000105 | 0.001931 | 0.000588 |
| MF | GO:0003678 | DNA helicase activity | 0.000224 | 0.001931 | 0.000588 |
| MF | GO:0003724 | RNA helicase activity | 0.000256 | 0.001931 | 0.000588 |
| MF | GO:0140097 | catalytic activity, acting on DNA | 0.002103 | 0.00888 | 0.002706 |

GO, Gene Ontology; BP, biological process; MF, molecular function.
