# Supplementary material for: Identification and experimental validation of common genes associated with both pulmonary arterial hypertension and major depressive disorder
Source: Front Psychiatry. 2025 Nov 7;16:1670519. doi: 10.3389/fpsyt.2025.1670519 (PMC12634556; doi:10.3389/fpsyt.2025.1670519)
Supplement: Supplementary file 2 [file DataSheet1.pdf]

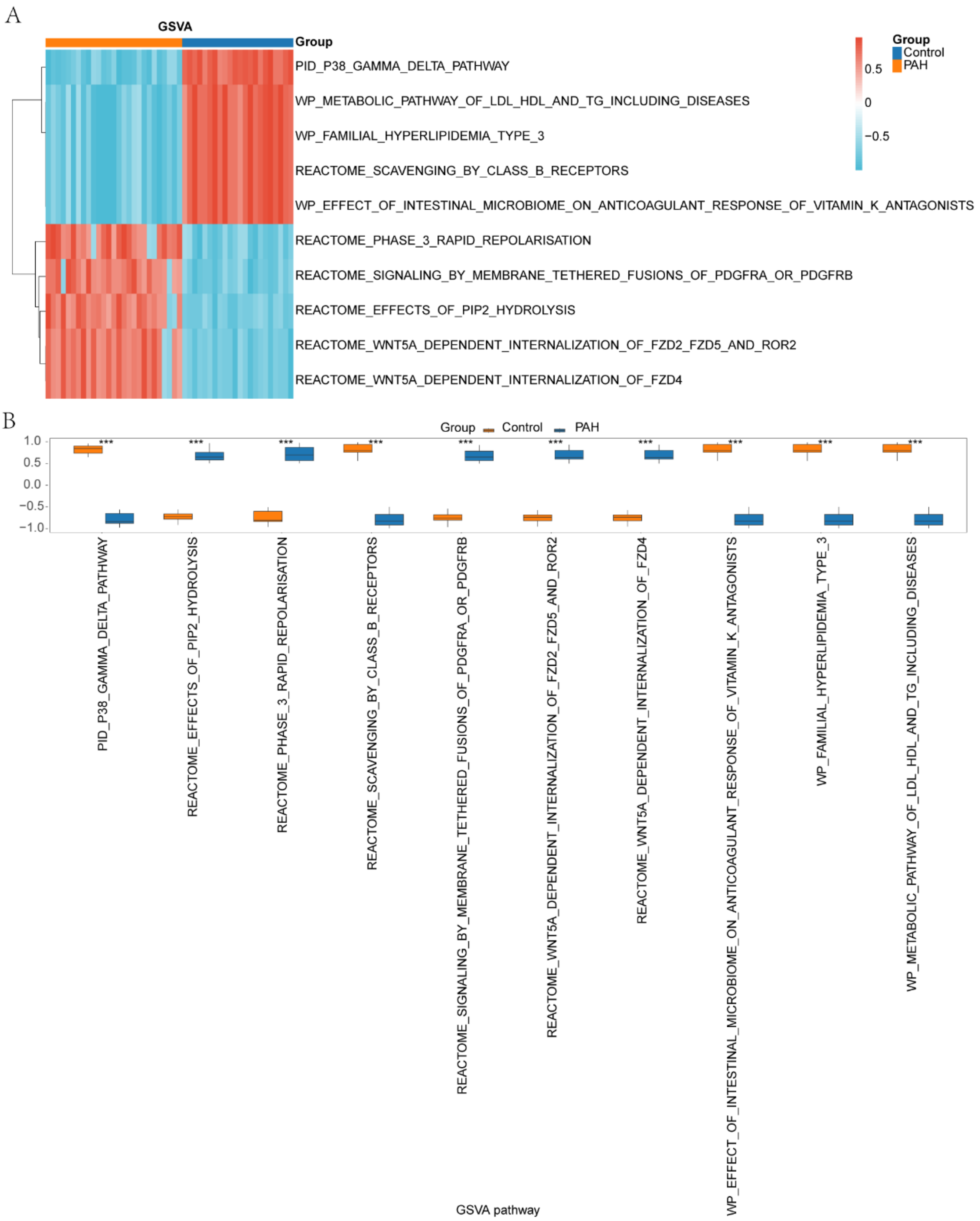

Fig. S1 Results of gene set variation analysis (GSVA) of the PAH dataset

A. A heatmap that visually represents the differences in gene set activity between the PAH and control groups within the PAH dataset. Each row in the heatmap represents a different gene set, and each column represents a sample. The color intensity within the heatmap indicates the level of activity of each gene set, with varying shades indicating higher or lower activity compared to the control. B. A bar chart highlighting gene sets with statistically significant differences ($p<0.05$) in activity between the PAH and control groups. Each bar represents a gene set, and the length of the bar indicates the extent of the difference in activity, with the direction indicating whether the activity is higher or lower in the PAH group compared to the control.

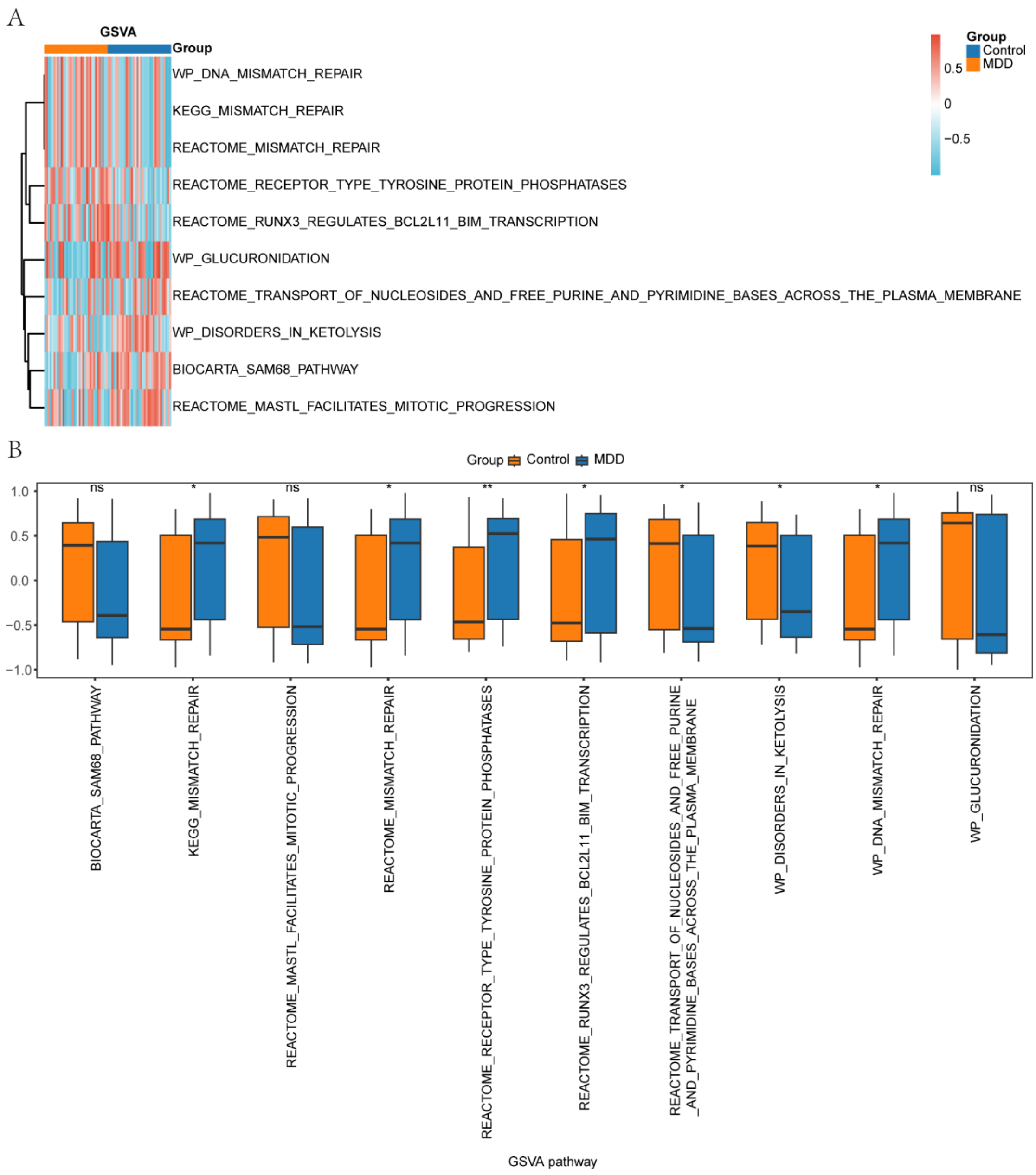

Fig. S2 Results of gene set variation analysis (GSVA) of the MDD dataset

A. A complex heatmap showing gene set differences between the MDD and control groups in the MDD dataset. B. A bar chart displaying gene sets with significant differences (p<0.05) between the MDD and control groups.

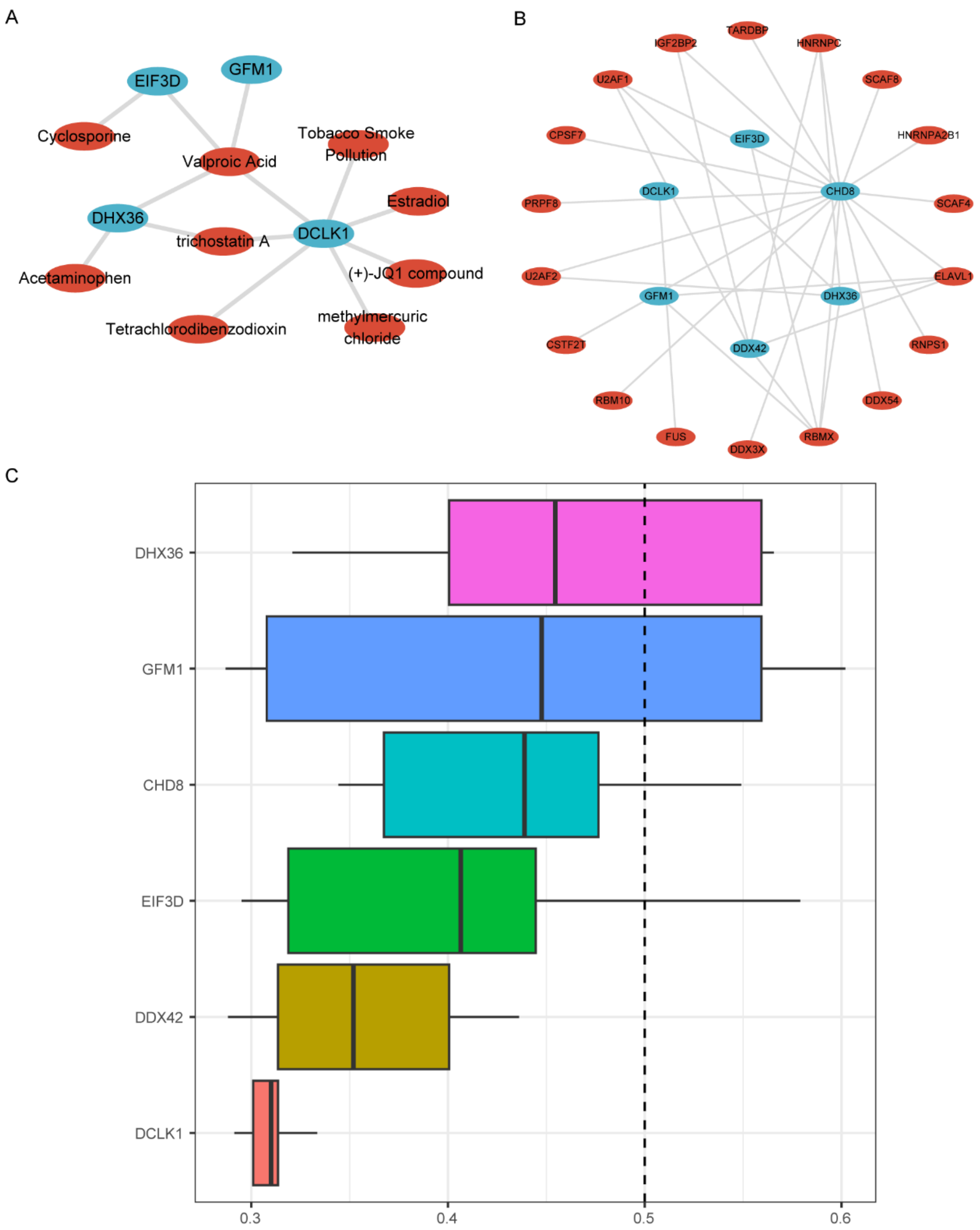

Fig. S3 Interaction network of common differentially expressed genes

A. The mRNA–drug network, mapping out the interactions between mRNAs of key genes and various drugs. B. The mRNA–RNA-binding protein network for key genes. RNA-binding proteins play crucial roles in the post-transcriptional regulation of gene expression, including splicing, transport, and stability of mRNA. C. Functional similarity analysis of key genes, illustrating how similar the functions of these genes are to each other.

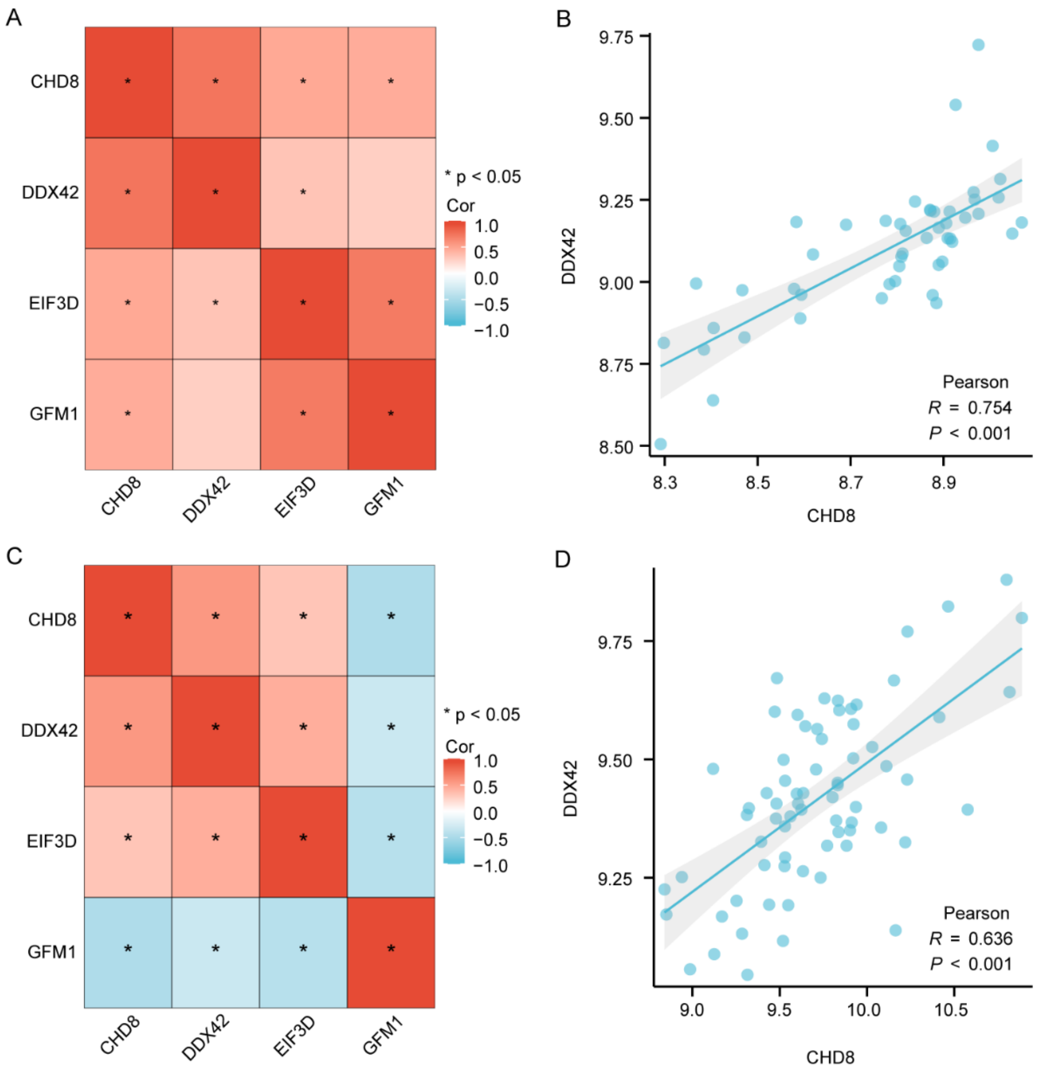

Fig. S4 Correlation analysis of key genes within the PAH and MDD datasets

A. Correlation heatmap for key genes in the PAH dataset. This heatmap displays the correlation coefficients between pairs of genes, with varying colors indicating the strength and direction of each correlation. Warmer colors typically represent stronger positive correlations, while cooler colors indicate negative correlations. B. Correlation scatter plot for the PAH dataset. C. Correlation heatmap for key genes in the MDD dataset, similar to panel A but focused on the MDD context. D. Correlation scatter plot for the MDD dataset. Following the example of panel B, this plot visualizes the correlation between two particularly correlated genes identified in panel C, helping to clarify the nature of their relationship in the context of MDD.

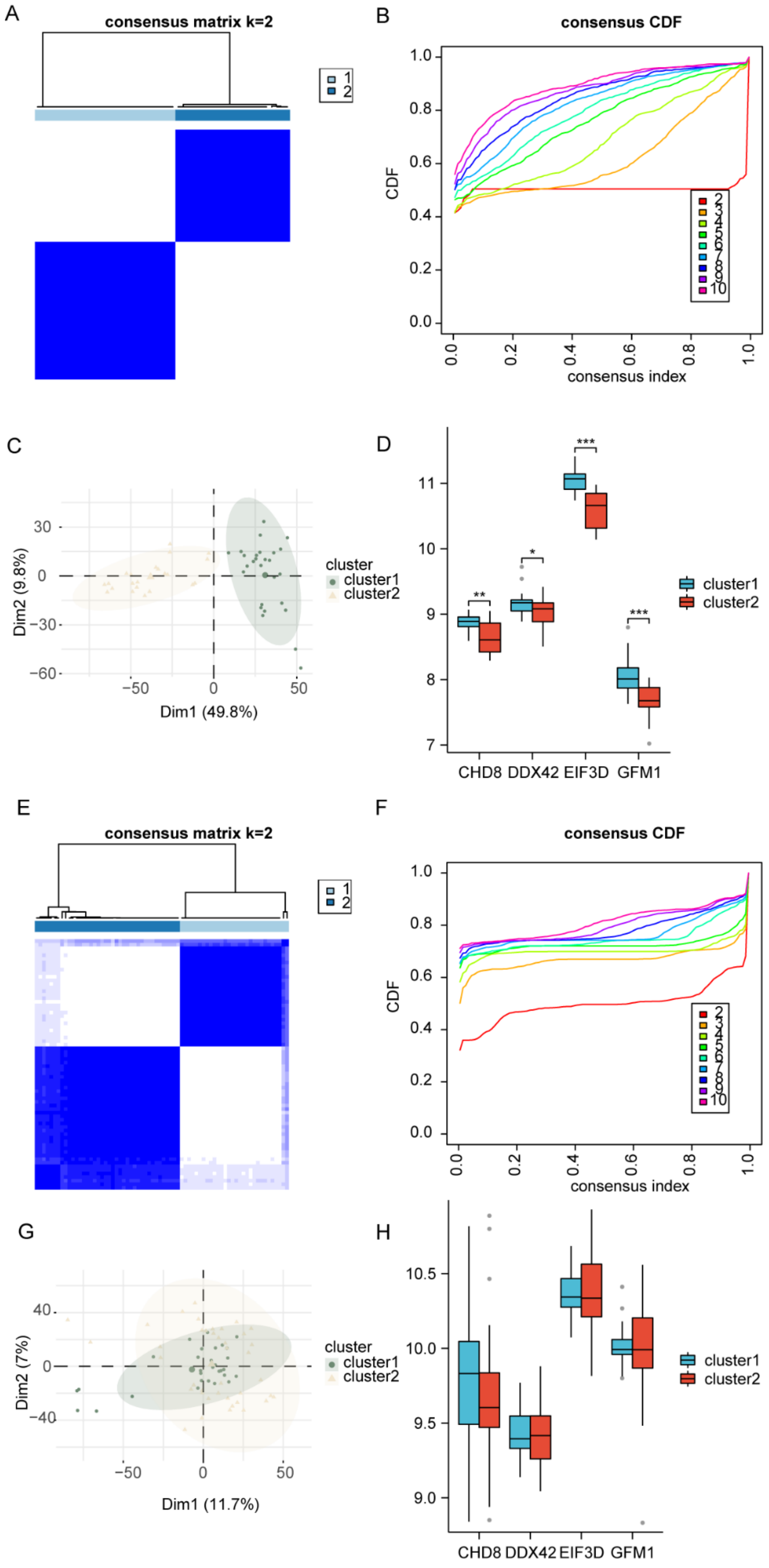

Fig. S5 Detailed overview of the consistent clustering results obtained from the

PAH and MDD datasets

A. The consistent clustering results for the PAH dataset. This panel shows how the samples from the PAH dataset are grouped into clusters based on their gene expression profiles, identifying potentially distinct subtypes within the PAH population. B. The cumulative distribution function (CDF) for the PAH dataset. The CDF plot helps determine the stability and reliability of the clustering results by showing the consistency levels across different numbers of clusters, aiding in the selection of the optimal cluster count. C. Principal component analysis (PCA) plots for cluster 1 and cluster 2 of the PAH dataset. These plots visualize the separation between the two clusters based on the principal components derived from the gene expression data, indicating how distinctly the data groups into these clusters. D. Box plots comparing the expression of hub genes between the two clusters in the PAH dataset. These plots highlight significant differences in gene expression levels between clusters, which may correspond to different disease phenotypes or severities. E. The clustering results based on the gene expression profiles within the MDD samples. F. The CDF for the MDD dataset, aiding in determining the optimal number of clusters by assessing the consistency of clustering. G. PCA plots for cluster 1 and cluster 2 of the MDD dataset, illustrating the separation and distinctiveness of these clusters as seen in the PCA analysis. H. Box plots for the MDD dataset, which compare the expression levels of hub genes between the two identified clusters, potentially reflecting different molecular mechanisms or disease states within MDD.

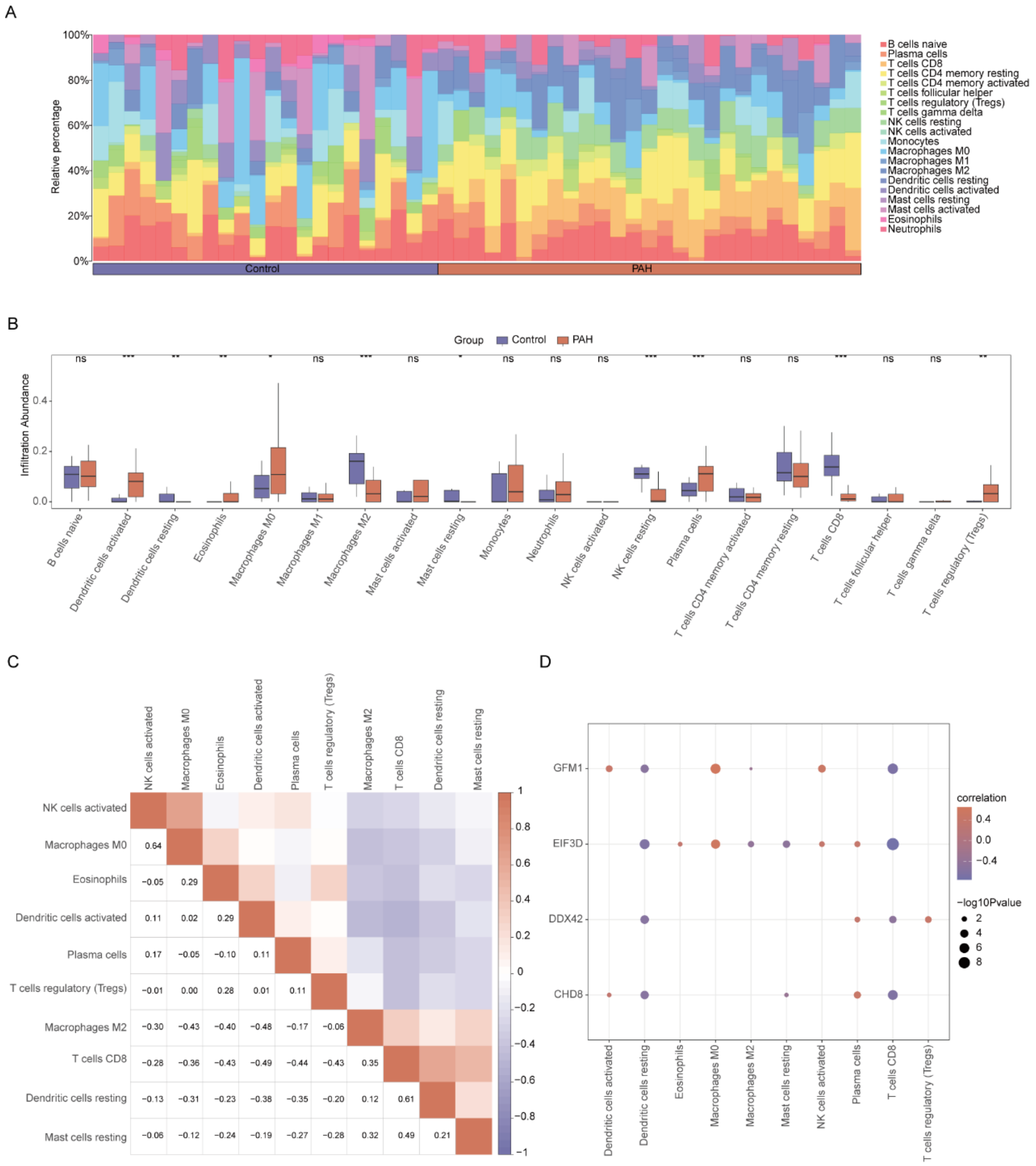

Fig. S6 Application of CIBERSORT analysis to evaluate the infiltration of immune cells in the PAH dataset

A. Stacked bar plots depicting the levels of 20 different immune cell types in the PAH dataset. Each bar represents a sample from the dataset, and the various colors within each bar denote the proportion of each immune cell type, providing a comprehensive overview of the immune cell composition in each sample. B. Comparison of immunocyte infiltration between the PAH and control samples. C. Heatmap of correlations among 10 significant immune cell types identified from the CIBERSORT analysis. The heatmap uses color coding to represent the strength and direction of the correlations, enabling quick identification of highly correlated or inversely related immune cell types, which might suggest cooperative or antagonistic relationships within the immune system in PAH. D. Spearman correlation analysis showing the relationships between four common core genes and the 10 significant immune cell types

identified in the PAH dataset. This analysis helps to elucidate how genetic factors might influence or be influenced by the immune microenvironment, potentially identifying gene–immune interactions that could be targeted for therapeutic benefit. Statistical significance indicators (ns, *, **, ***, ****) are used throughout the figure to denote the levels of statistical significance.

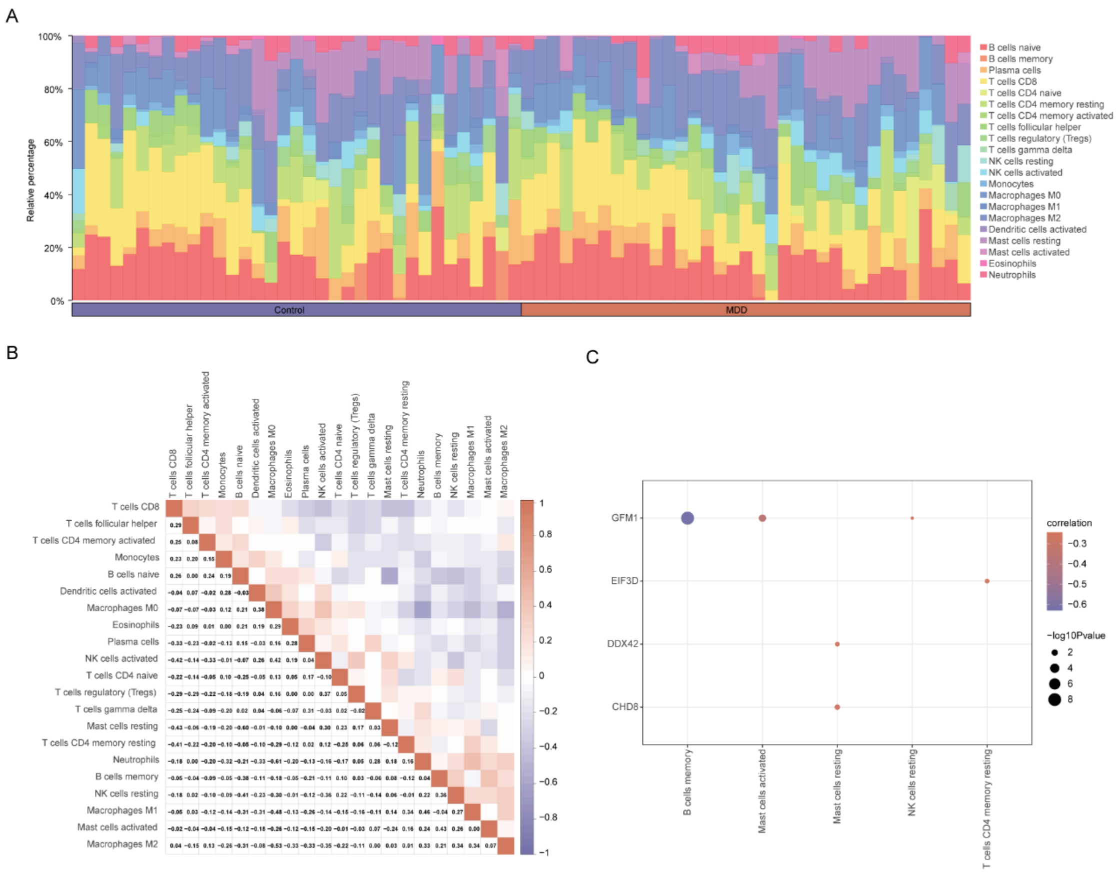

Fig. S7 Results from the CIBERSORT analysis used to assess the infiltration of immune cells within the MDD datasets

A. Stacked bar plots that illustrate the relative levels of 21 types of immune cells across the MDD dataset. Each bar represents a different patient sample, and the various colors in each bar indicate the proportion of each immune cell type, providing a visual breakdown of immune cell distribution and diversity in MDD patients compared to controls. B. Heatmap of correlation among the 21 types of immune cells identified in the MDD dataset. C. Spearman correlation analysis between four common core genes and the 21 types of immune cells. Statistical significance indicators (ns, *, **, ***, ****) are applied throughout the figure to highlight the statistical relevance of the findings, marking nonsignificant to highly significant correlations and differences.
